# Bone or Tooth dentin: The TGF-β signaling is the key

**DOI:** 10.7150/ijbs.97206

**Published:** 2024-06-24

**Authors:** Chunmei Xu, Xudong Xie, Yafei Wu, Jun Wang, Jian Q. Feng

**Affiliations:** 1 Shanxi Medical University School and Hospital of Stomatology, Shanxi Province Key Laboratory of Oral Diseases Prevention and New Materials, Taiyuan 030001, China.; 2 State Key Laboratory of Oral Diseases, National Center for Stomatology, National Clinical Research Center for Oral Diseases, Department of Periodontics, West China Hospital of Stomatology, Sichuan University, Chengdu 610041, Sichuan China.

**Keywords:** Cell lineage tracing, tooth development, cell fate switch, Gli1-CreERT2, 3.2 kb Col1-CreERT2, evolution

## Abstract

To investigate the cell linkage between tooth dentin and bones, we studied TGF-β roles during postnatal dentin development using *TGF-β receptor 2* (*Tgfβr2*) cKO models and cell lineage tracing approaches. Micro-CT showed that the early *Tgfβr2* cKO exhibit short roots and thin root dentin (n = 4; p<0.01), a switch from multilayer pre-odontoblasts/odontoblasts to a single-layer of bone-like cells with a significant loss of ~85% of dentinal tubules (n = 4; p<0.01), and a matrix shift from dentin to bone. Mechanistic studies revealed a statistically significant decrease in odontogenic markers, and a sharp increase in bone markers. The late *Tgfβr2* cKO teeth displayed losses of odontoblast polarity, a significant reduction in crown dentin volume, and the onset of massive bone-like structures in the crown pulp with high expression levels of bone markers and low levels of dentin markers. We thus concluded that bones and tooth dentin are in the same evolutionary linkage in which TGF-β signaling defines the odontogenic fate of dental mesenchymal cells and odontoblasts. This finding also raises the possibility of switching the pulp odontogenic to the osteogenic feature of pulp cells via a local manipulation of gene programs in future treatment of tooth fractures.

## Introduction

The onset of a bone-like dentin structure (osteo-dentin) is known of in injured or diseased or gene knocked-out mice 1-6. Fossil studies have provided emerging evidence: the origin of bones before teeth (meaning dentin), which suggests that bone and tooth formation (meaning dentin) are closely related 7-11. However, there is a lack of genetic and cellular evidence to show a direct linkage between bones and dentin during development or tooth-repairing processes.

Bones and dentin share many features in structures, mineral character, and cellular characteristics except for the following two key differences: 1) dentin have unique dentinal tubules but bones do not 12, 13; and 2) fractured teeth cannot be repaired by themselves 14 but fractured bone can be restored via their endogenous remodeling mechanisms 1. Thus, the fractured teeth can only be either surgically extracted or “glued” using adhesive materials 6.

Crown dentinogenesis starts from the embryonic stage, while root dentinogenesis begins postnatally 15-17. Interestingly, the odontoblasts (Od) of crown and roots are derived from dental papilla mesenchymal cells and are responsible for the synthesis, secretion, and mineralization of dentin extracellular matrices 18, 19, the crown and root formation were selectively regulated by different genes 20.

It is known that superfamily members of TGF-β (transforming growth factor-beta) are highly conserved in organisms ranging from Nematoda and Arthropoda to Mammalia, which are involved in regulating animal body plans such as anteroposterior, dorsoventral (bilateral), left-right axes, individual organs, and regulation of tissue development and homeostasis 21, 22. Expression of type II TGF-β receptor (TGF-βR2) in craniofacial progenitor cells, dental pulp cells and odontogenic lineage cells has been well documented *in vitro* and* in vivo*. For example, during dentinogenesis, TGF-βR2 is continuously expressed throughout the functional differentiation and maturation of dental progenitor cells into odontoblasts, which contribute to odontogenesis predominantly via binding to different TGF-β ligands 23-26. Blocking of the signaling mediated by TGF-βR2 largely suppress dentinogenesis 27. Conventional *Tgfβr2* knockout (KO) studies using non-inducible Cre lines showed tooth defects, including ectopic bone formation in tooth pulp 26, 28, 29. These studies raise a hypothesis: the TGF-β signaling pathway controls the cell linkage between bones and dentin.

In this study we attempted to test the following three hypotheses beyond simple characterizations of gene knockout phenotypes: 1) whether bones and dentin are in the same cell lineage; 2) specific roles of TGF-β signaling during postnatal development of crown and root dentin; 3) how TGF-β signaling defines the fate of pulp cells. To achieve these goals, we targeted *Tgfβr2* at different developmental stages using two specific tamoxifen induced-Cre mouse lines: the G*li1-Cre^ERT2^* line targeting the root pulp cells, and the *3.2-kb Col1-Cre^ERT2^* (targeting the crown pulp and odontoblasts) combined with cell-lineage tracing approaches. Our studies for the first time showed that the loss of TGF-β signaling leads to a replacement of odontoblasts by osteoblast-like cells and the switch from dentin-to-bone matrices, supporting the same cell-lineage linkage between odontoblasts (for forming dentin) and osteoblasts (for forming bone). This finding also raises the possibility of switching the pulp cells from odontogenic to osteogenic via local manipulation of gene programs in future treatment of tooth fractures.

## Materials and Methods

### Transgenic mice breeding and sample collection

All experimental protocols followed ARRIVE (Animal Research Reporting of *In vivo* Experiments) guidelines and were approved by the Animal Care and Use Committees (IACUC) at Texas A&M University College of Dentistry.

*Gli1^CreERT2/+^*, *3.2-kb Col1^CreERT2/+^*, *R26R^tdTomato/+^*(stock number: 007905), *Tgfβr2^flox/+^* (stock number: 012603) mice were purchased from Jackson Laboratory and housed in a temperature-controlled environment with 12-h light/dark cycles. To trace the Gli1^+^/ 3.2-kb Col1^+^ lineage cells during postnatal dentin development, the *Gli1^CreERT2/+^* mice and *3.2-kb Col1^CreERT2/+^* mice were crossed with *R26R^tdTomato/+^* reporter mice respectively. The* Gli1^CreERT2/+^*,* R26R^tdTomato/+^* mice were then crossed with *Tgfβr2^flox/+^* mice to get *Gli1^CreERT2/+^*,* R26R^tdTomato/+^*,* Tgfβr2^flox/+^* mice. Next, the *Gli1^CreERT2/+^*,* R26R^tdTomato/+^*,* Tgfβr2^flox/flox^* mice were generated by crossing *Gli1^CreERT2/+^*,* R26R^tdTomato/+^*,* Tgfβr2^flox/+^* mice with *Tgfβr2^flox/+^* mice. The same strategy was applied to generate *3.2-kb Col1^CreERT2/+^*,* R26R^tdTomato/+^*,* Tgfβr2^flox/flox^* mice. Because no apparent difference was detected among the studied animals of different genders, both male and female mice were used in this study. Tamoxifen (75 mg/kg body weight) was administrated at postnatal day 5 (P5). Mice were harvested at P6, P14 for lineage tracing experiments and P28 for comparing the effect of removing *Tgfβr2* on postnatal dentinogenesis. Mandibles were fixed in freshly prepared 4% paraformaldehyde (PFA) and decalcified in 10% ethylenediaminetetraacetic acid (EDTA) for future use.

### Micro-computed tomography (μ-CT)

Mandible μ-CT analysis was performed by Scanco μ-CT 35 (Scanco Medical, Bassersdorf, Switzerland), and three-dimensional reconstruction was carried out with Imaris 9.0 (Bitplane) as previously described 30.

### Double-fluorochrome labeling

Calcein and Alizarin red were administered separately (five days apart) at before harvest. Fixed mandibles were embedded in methyl-methacrylate (MMA, Buehler, Lake Bluff, IL). Sample blocks were cut and polished. Fluorescence scanning was performed to analyze dentin mineral deposition as previously reported 30, 31.

### Histological analysis and Immunostaining

Mandibles were embedded in paraffin using standard histological procedures 30. Decalcified samples for cell lineage tracing were dehydrated with 30% sucrose and embedded in OCT followed by CryoJane frozen sections 2. Five-μm-thick sections were collected for hematoxylin and eosin (H&E), Masson's trichrome and Sirius red staining. Immunostaining was carried out as previously described^31^ with following primary antibodies: anti-DSPP mouse antibody (provided by Dr. Chunlin Qin at Texas A&M University College of Dentistry, 1:400), anti-NESTIN mouse antibody (MAB353, Millipore Sigma, 1:200), anti-DMP1 rabbit polyclonal antibody (generously provided by Dr. Chunlin Qin, Texas A&M University College of Dentistry; 1:200), anti-Osterix (OSX) rabbit antibody (ab22552, abcam, 1:200), anti-Collagen I rabbit antibody (ab21286, abcam, 1:200). anti-Ki67 rabbit antibody (ab16667, abcam, 1:200), anti-PCNA rabbit antibody (Cst13110s, Cell signaling technology, 1:200), anti-β-catenin mouse antibody (DSHB-PY489, Developmental Studies Hybridoma Bank, 1:50).

### Scanning electron microscopy

Mandibles were fixed in 4% PFA, dehydrated and embedded in MMA. The MMA-embedded samples were cut, polished and treated with 37% phosphoric acid 31. Air-dried samples were coated with gold and palladium, and a JEOL JSM-6300 scanning electron microscope (JEOL Limited, Tokyo, Japan) was used to perform the analyses as reported previously 31, 32.

### RNAscope® assay

Freshly harvested mandibles were fixed in 10% formalin for 24 hours at room temperature and decalcified in 10% EDTA at 4℃. Well-decalcified samples were embedded in paraffin and cut. Five-μm-thick sections were used and RNAscope® assay was performed following RNAscope®2.5 BROWN (Advanced Cell Diagnostics, 322300, 322310) for FFPE manufacturer's protocol^33^ with following RNA probes: Positive Control Probe (Advanced Cell Diagnostics, 313911), Negative Control Probe (Advanced Cell Diagnostics, 310043), Mm-Dspp (Advanced Cell Diagnostics, 448301), Mm-Dmp1 (Advanced Cell Diagnostics, 441171), Mm-Osx (Advanced Cell Diagnostics, 403401) , Mm-β-catenin (Advanced Cell Diagnostics, 537601). Images and the semi-quantification were analyzed using ImageJ software 34 based on the RNAscope scoring categories (Table S1).

### Statistical analysis

Data are shown as box-and-whisker plots (with median and interquartile ranges) from max to min, with all data points shown. Analyses were performed by an independent sample t-test for parametric analysis, and Mann-Whitney test was used for non-parametric analysis using SPSS 19.0 (SPSS Inc, Chicago, IL). A P value < 0.05 was considered statistically significant.

## Results

### Ablation of *Tgfβr2* in Gli1^Lin^ cells leads to delayed 3^rd^ molar eruption and abnormality in postnatal dentinogenesis, especially in root dentin formation

**Figure 1:**
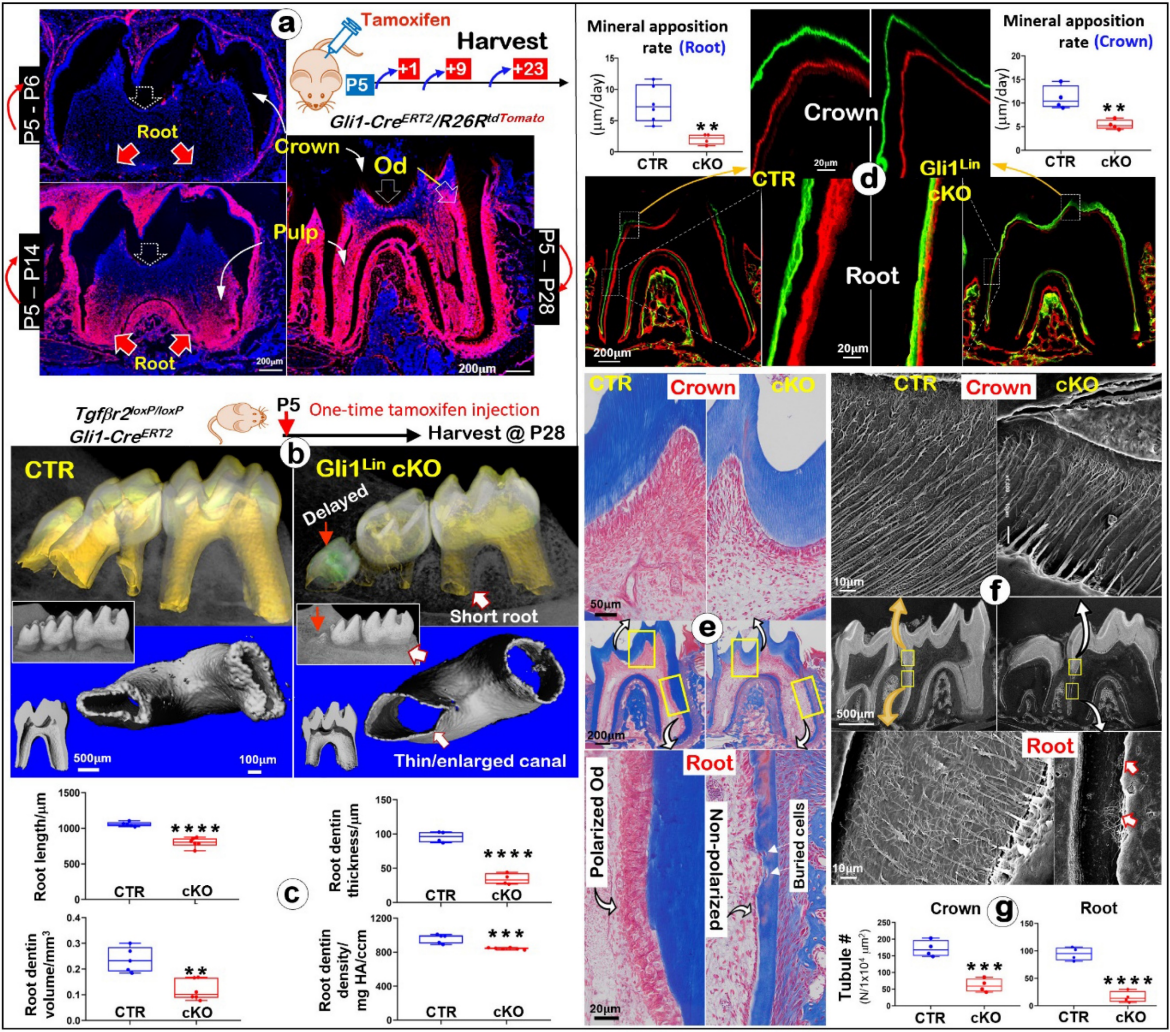
** Lineage tracing of Gli1^Lin^ tdTomato^+^ cells in the root pulp, and development of molar root defects in Gli1^Lin^ cKO including, impaired mineralization, changes of Od cell morphologies, and the loss of dentinal tubules. a.** Lineage tracing of Gli1^Lin^ cells in root formation. *Gli1*^CreERT2/+^, *R26R^tdTomato/+^* mice were administrated one dose of tamoxifen at postnatal day 5 (P5) and followed by 3 chasing timepoints: 1-, 9-, and 23-days post injection, separately.** b.** One-time injection of tamoxifen (administered at P5 and harvested at P28) induced severe tooth root defects in Gli1^Lin^ cKO (right panels) by representative Micro-computed tomography (μCT) analyses; and** c.** quantitation of changes of root length, dentin thickness, and mineral density (n = 4-6; **P< 0.01; ***P< 0.001; ****P< 0.0001)**; d**. Representative Calcein (green color) and alizarin red (red color) double injections (5 day apart) revealed drastic reduction in mineral apposition rate in the Gli1^Lin^ cKO root (lower panel) and crown (upper panel). Quantitation data showed that these changes were statistically significant (n = 4-6; **P< 0.01); **e**. Representative Masson's Trichrome staining images showed expanded root canals and thin dentin plus a replacement of postulated polarized odontoblasts by a single layer of non-polarized in the Gli1^Lin^ cKO root (right panels);** f.** Acid-etched SEM images displayed a lack of dentin tubules in the Gli1^Lin^ cKO root (lower *right*) and a moderate reduction in the Gli1^Lin^ cKO crown dentin tubules (*upper right*); g. Statistic data analyses showed that those changes were significant (n=4; ***P< 0.001; ****P< 0.0001). Od, odontoblast.

Gli1-Cre^ERT2^ is a useful mouse tool to target dental mesenchymal progenitor cells during tooth development 35, 36, although its activity in crown and root during postnatal development is unclear. Thus, we first examined the Gli1-Cre^ERT2^ labeling pattern in molars with *Gli1^CreERT2/+^*/*R26R^tdTomato/+^* mice. One-time tamoxifen induction at postnatal day 5 (P5) displayed few tdTomato^+^/ red cells at P6 but many red-cells in the root pulp at P14 (**Fig. 1a**). By P28 there were massive red pulp cells and odontoblasts (Ods) in roots with some signals in crown (**Fig. 1a**), indicating a high Cre activity in the postnatal root with a weak expression in the crown.

Next, we generalized the Gli1^Lin^ *Tgfβr2* cKO line by crossing *Gli1^CreERT2/+^*,* R26R^tdTomato/+^*,* Tgfβr2^flox/flox^*, followed by one-time injection of tamoxifen at P5 and harvested at P28 (**Fig. 1b**). All mutant pups showed no apparent gross defects. The representative μCT images of the Gli1^Lin^ cKO molar displayed a delay-erupted 3^rd^ molar (red solid arrow), short and thin-wall molar roots plus enlarged root canals (**Fig. 1b**). Quantitative analyses in **Fig. 1c** showed that in comparison with control molars, the Gli1^Lin^ cKO root length was ~20% shorter (P<0.0001), the root dentin thickness was decreased by ~65% (P<0.0001), the root dentin volume was ~50% reduced (P<0.01), plus a significantly lower dentin density (P<0.001). However, compared to the control crown, there were only minor changes in the Gli1^Lin^ cKO crown as shown in supplementary Figure 1 (**Figure S1**), including a minor reduction in crown dentin volume (~10%, P<0.05), and a moderate decrease in dentin density (~5%, P<0.01).

To define changes of mineral apposition rates, Calcein and Alizarin red injections were performed before the harvest (5 days apart). The confocal images showed ~75% reduction in the apposition rate in the Gli1^Lin^ cKO root (P<0.01) and significant reduction in the crown (P<0.01) compared to the control (**Fig. 1d**). Histological analyses using Masson's trichrome staining showed osteocyte (Ocy)-like cells embedded in the poorly formed dentin matrix of the Gli1^Lin^ cKO root, and a replacement of polarized tall-columnar odontoblasts by a single layer of non-polarized osteoblast (Ob)-like cells (**Fig. 1e**). On the other hand, there were no apparent changes in the Gli1^Lin^ cKO crown (**Fig. 1e**). SEM images showed that, in comparison with control, the number of dentinal tubules were sharply decreased in both crown and root in the Gli1^Lin^ cKO (**Fig. 1f-g**), which was statistically significant (P<0.001 and P<0.0001, respectively). In addition, the Gli1^Lin^ cKO root canal was significantly wider than control (**Figure S2**).

Together, these findings clearly demonstrated that the early removal of *Tgfβr2* leads to the loss of root dentin tubules, inhibitions in mineral apposition, and a switch from Od-like to Ob-like cells.

### Removing *Tgfβr2* in molar roots changes dentin matrix features and molecular expressions from the odontogenic-to-osteogenic features

**Figure 2:**
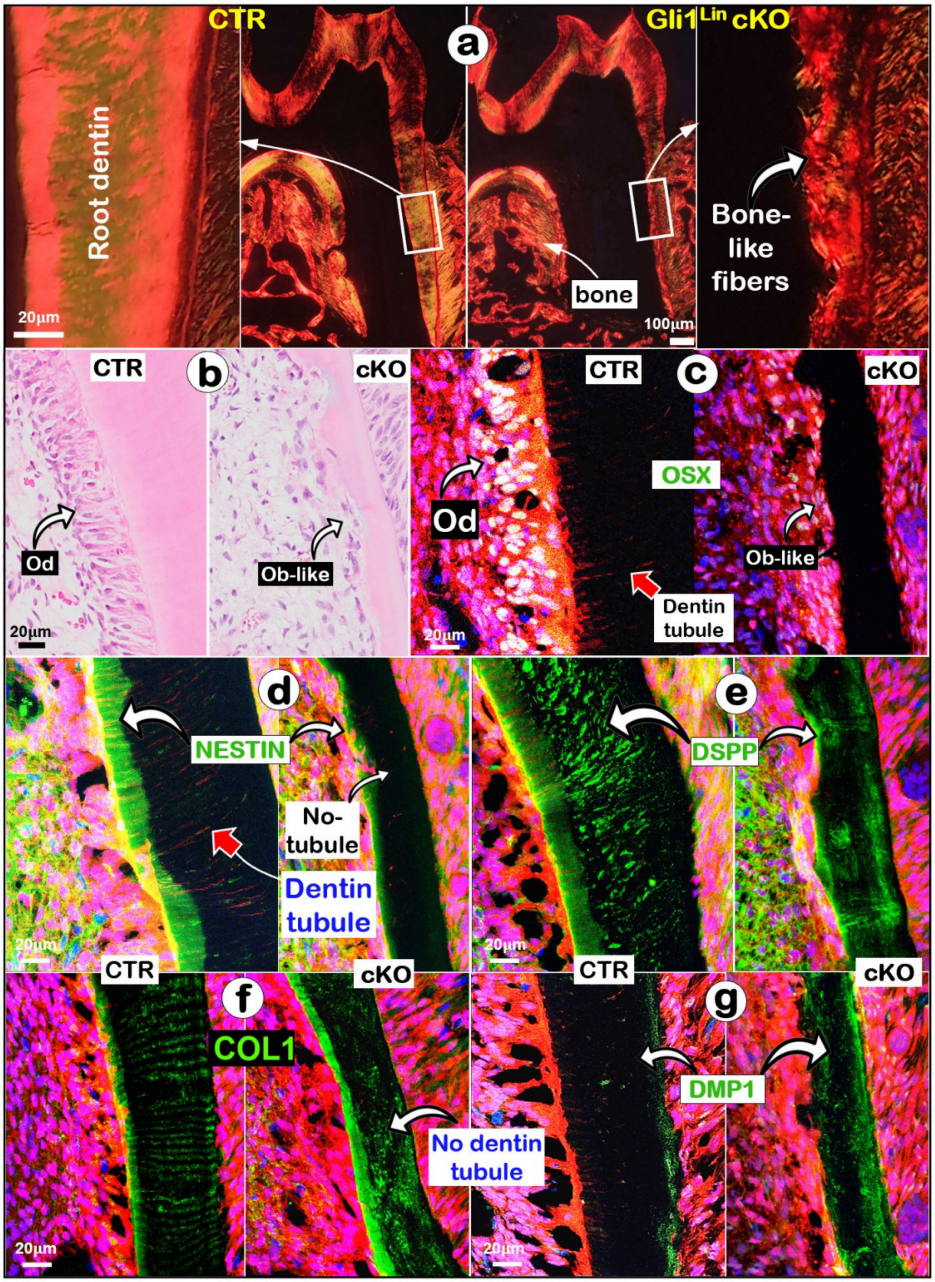
** Defected dentin matrices, a replacement of polarized odontoblasts (Ods) by a flat layer of osteoblast (Ob)-like cells, and molecular changes occurred in the Gli1^Lin^ cKO roots. a.** Representative polarized light images displayed a disorganized dentin fiber with no sign of dentin tubules;** b.** H&E images showing a flat layer of Ob-like cells in the Gli1^Lin^ cKO roots; and** (c-g)** Immunofluorescence staining images showed the following changes in the Gli1^Lin^ cKO root: a sharp decrease in OSX with no sign of polarized cells (**c**); a great reduction of NESTIN in pre-dentin and a lack of dentin tubules (**d**); disperse DSPP distribution (**e**) and COL1 staining (**f**) in the Gli1^Lin^ cKO roots; and a drastic increase in DMP1 in the Gli1^Lin^ cKO root dentin (**g**).

To address why sharp changes of the Gli1^Lin^ cKO root dentin structure took place, we first showed a change from a smooth fiber organization pattern to an irregular bone-like fiber arrangement in the Gli1^Lin^ cKO (**Fig. 2a**). Next, we documented a replacement of polarized Ods by Ob-like cells using H&E staining (**Fig. 2b**). Third, our immunofluorescent images showed that OSX, an essential transcriptional factor for postnatal tooth root but not for crown formation 37, was highly expressed in multi-layers of pre-odontoblasts and polarized Ods in control, but were sharply reduced in the Gli1^Lin^ cKO (**Fig. 2c**). Furthermore, representative images of NESTIN displayed a high expression level in the control predentin but a weak expression in the Gli1^Lin^ cKO predentin (**Fig. 2d**). Similarly, high levels of DSPP (a non-collagenous matrix protein essential for odontoblast maturation and dentin formation) 38 (**Fig. 2e**) and type 1 collagen (COL1;** Fig. 2f**) were present with the root dentin tubules in control, while a disperse expression pattern was observed in Gli1^Lin^ cKO. Finally, there was a great increase of DMP1 (a classic bone marker highly expressed in osteocytes) 32 in the Gli1^Lin^ cKO root dentin (**Fig. 2g**).

**Figure 3:**
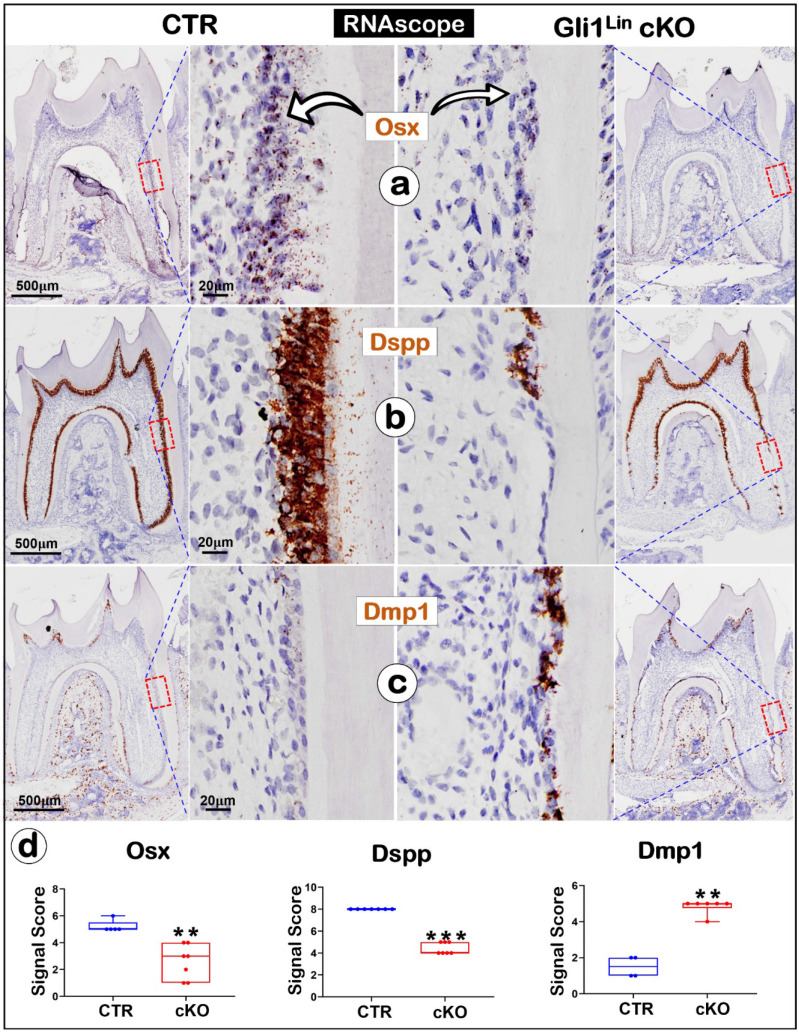
** The RNAscope data analyses revealed a switched mRNA expression profile characterized with increased osteogenic markers and decreased odontogenic markers in the Gli1^Lin^ cKO root compared to the control**. **a**) A drastic decrease of *Osx* mRNA levels in the Gli1^Lin^ cKO root *(right panel)*; **b**) A great decrease or lack of *Dspp* expression in Gli1^Lin^ cKO Ob-like cells; and **c**) an increase in *Dmp1* mRNAs in the Gli1^Lin^ cKO Ob-like cells; and **d**) Statistical data analyses revealed the above changes were statistically significant (n = 4~7; **P< 0.01; ***P< 0.001). Ob, osteoblast.

We also studied the mRNA changes of the key molecules using the RNAscope technique 33. For example,* Osx* and* Dspp* are sharply reduced (**Fig. 3a-b**), whereas *Dmp1* was greatly upregulated (**Fig. 3c**). Quantitation data further confirmed that these changes are statistically significant (**Fig. 3d**). Of note, both the negative (**Figure S3a**) and positive control data (**Figure S3b**) showed the tissue specificity and a low “background noise” of this technique.

The above findings revealed significant reductions of *Osx, Dspp* but a sharp increase of *Dmp1* in the Gli1^Lin^ cKO root, which supports a vital role of TGF-β signaling in controlling the odontogenic cell fate.

### Deletion of *Tgfβr2* in the 3.2-kb Col1^Lin^ leads to a thin crown, a replacement of Ods by Ob-like cells, and the onset of ectopic bones in the crown pulp

**Figure 4:**
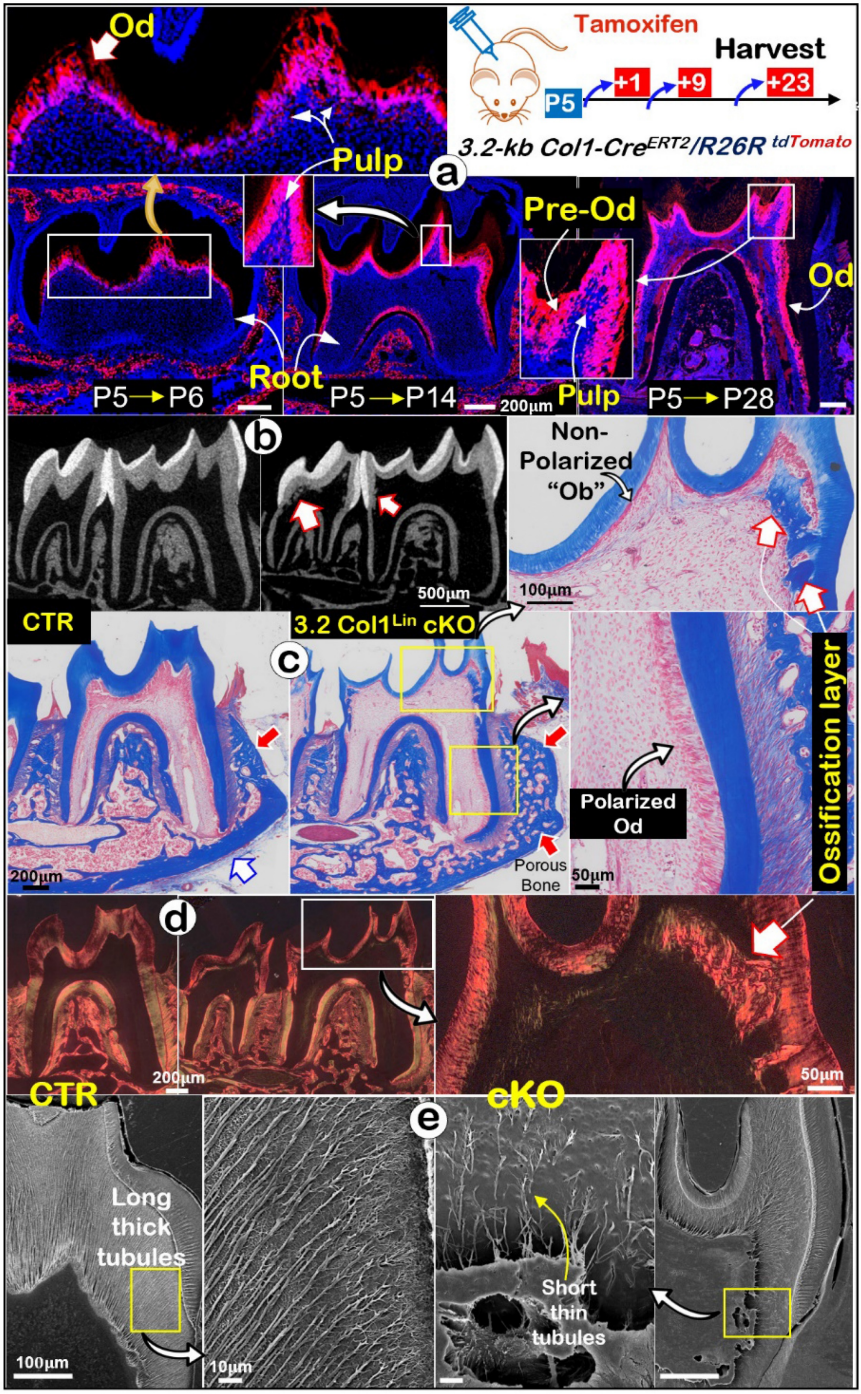
** Lineage tracing of 3.2 Col1^Lin^ *tdTomato^+^* cells and tooth crown defects in 3.2 Col1^Lin^ cKO**. **a**. Lineage tracing of 3.2 Col1^Lin^ cells in crown (dominant) and root. *3.2-kb Col1^CreERT2/^****^+^***, *R26R^tdTomato/+^* mice were administrated 1 dose of tamoxifen at postnatal day 5 (P5) and followed by 3 chasing timepoints: 1, 9, and 23 days, separately.** b**. One-time injection of tamoxifen (@P5 and harvested at P28) induced remarkable tooth crown phenotypes in 3.2 Col1^Lin^ cKO (right panels) were revealed by representative μCT analyses, in which red open arrows pointed ectopic ossification in the crown pulp; **c**. Masson's Trichrome staining showed thin crown dentin and non-polarized Ob-like cells plus ectopic ossification in the pulp chamber; **d**. Polarized light microscope images revealed irregular fiber organization in the ectopic ossification tissue; and **e.** SEM images displayed short/thin dentin tubules in the 3.2 Col1^Lin^ cKO crown (also see Figure S7 for abnormal changes of root dentin tubules and quantitation data of changes of the tubule numbers). Od, odontoblast; Pre-od, pre-odontoblasts; Ob, osteoblast.

It's known that molar crown develops ahead of roots 20 and the Gli1^Lin^ is largely inactive during development of postnatal crowns (**Fig. 1a**). To effectively target *Tgfβr2* in molar crown Ods, we studied the expression pattern of 3.2-kb Col1^Lin^ (a classic bone cell line” 39). The one-time tamoxifen induction at postnatal day 5 (P5) led to the restricted label in crown Ods plus some crown pulp cells at P6 (**Fig. 4a***, upper*). By P14 and P28, strong red signals were detected in all Ods and some pulp cells of the crown and root Ods (**Fig. 4a***, lower*). Next, we crossed this line to *Tgfβr2^flox/flox^* and *R26R^tdTomato/+^* lines for generation of the 3.2 Col1^Lin^ cKO with one-time induction at P5 and harvested at P28.

The representative μCT images showed thin crown dentin, widened pulp chamber and ectopic ossification in the 3.2 Col1^Lin^ cKO crown pulp plus a thin root dentin (**Fig. 4b, Figure S4a**). The quantitative results showed significantly reduced dentin volume in the 3.2 Col1^Lin^ cKO crown (~30%, P<0.001) and root (~30%, P<0.05). There was a minor reduction in the 3.2 Col1^Lin^ cKO root length (P<0.05) and ~40% reduction in the root dentin thickness compared to the control (**Figure S4b;** P<0.01). Interestingly, there was no statistically significant difference between the control and the 3.2 Col1^Lin^ cKO in root dentin density (**Figure S4b**). Of note, the double labeling data showed striking ectopic ossification in the cKO crown (**Figure S5a**) but there was no apparent change in mineral apposition rates in the cKO root (**Figure S5b**).

Masson's trichrome stains (**Fig. 4c** and **Figure S6a**) displayed a sharp reduction in the crown dentin thickness, a replacement of polarized Ods by a single layer of flat Ob-like cells, and massive ectopic ossification in which bone-like cells were embedded in the 3.2 Col1^Lin^ cKO. On the other hand, there were no apparent changes in the 3.2 Col1^Lin^ cKO root Ods (**Fig. 4c**). The polarized light microscopic images showed bone-like fibers in the ectopic ossification tissue distinct from the classic dentin matrix (**Fig. 4d** and **Figure S6b**)*.* Sirius Red staining showed that the 3.2 Col1^Lin^ cKO-caused ectopic osteo-dentin was surrounded by Ob- and Ocy-like cells (**Figure S6c**).

SEM images revealed that, in comparison with the control, the number and length of dentinal tubules were greatly decreased in the 3.2 Col1^Lin^ cKO crown with a moderate change in the 3.2 Col1^Lin^ cKO root (**Fig. 4e** and **Figure S7a**). The quantitative SEM data displayed ~75% reduction of dentin tubule number in the 3.2 Col1^Lin^ cKO crown (P<0.001) vs ~50% reduction in the 3.2 Col1^Lin^ cKO root (P<0.05) (**Figure S7b**). We also noticed that the entrapped cells in the ectopic bone-like tissue have multiple cellular processes similar to osteocytes (**Figure S8**), further supporting the cell switch from the odontogenic to the osteogenic lineage.

In summary, the removal of *Tgfβr2* in molar crown/odontoblasts by the *3.2-kb Col1^CreERT2/+^* line results in a similar cell fate change as observed in the Gli1^Lin^ cKO plus the onset of massive ectopic ossification in the crown pulp.

### Ablation of* Tgfβr2* in molar crowns alters expressions of molecules necessary for maintaining the odontogenic features

**Figure 5:**
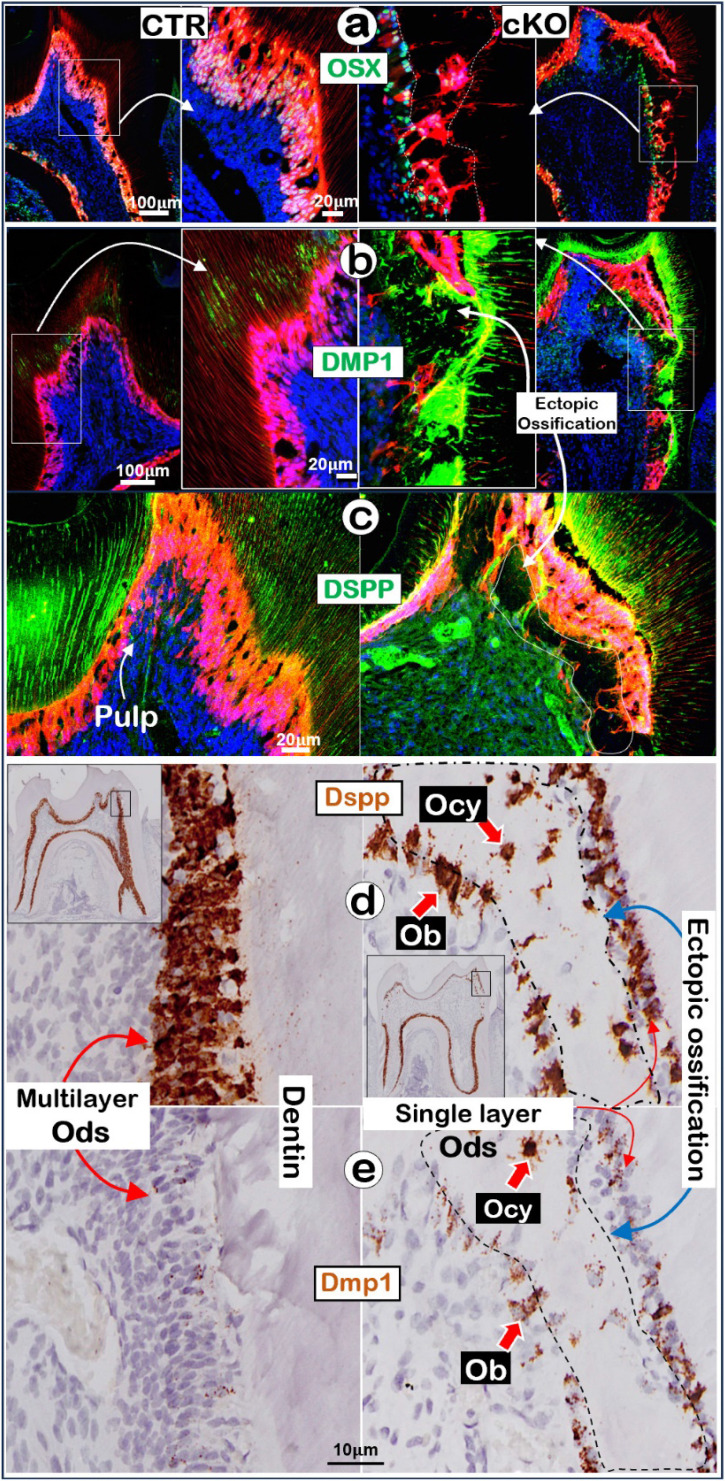
** Removal of *Tgfβr2* caused ectopi*c* ossification in the crown pulp with molecular changes in the 3.2 Col1^Lin^ cKO crown**. **a-c.** Representative immunofluorescent images showed a great decrease of OSX in the 3.2 Col1^Lin^ cKO crown cells plus a high expression level in the Ob-like cells along the ossification cite (**a**); sharp increase of DMP1 in the ossification site (**b**) and reduced DSPP expression.; **d-e**. The RNAscope images revealed a decrease of *Dspp* expression in Ods of 3.2 Col1^Lin^ cKO but an ectopic expression in the Ob-like and Ocy-like cells (**d**), and an increase in *Dmp1* mRNAs in the Ods and Ob-like cells of 3.2 Col1^Lin^ cKO (**e**). Ob, osteoblast; Ocy, osteocyte; Od, odontoblast.

To further understand the molecular mechanism by which Ods switch to Ob-like cells in the 3.2 Col1^Lin^ cKO crown, we first showed a high level of OSX in polarized multilayer-Ods in the control vs a few single-layer-OSX^+^ Ob-like cells on the surface of ossified tissues in the cKO crown (**Fig. 5a**). Next, our immunofluorescent images displayed a high level of DMP1 (**Fig. 5b**) but a low level of DSPP in the cKO crown (**Fig. 5c**). Furthermore, the RNAscope data showed a decrease in *Dspp* in the Ods but a relative high level of *Dspp* in the Ob-or Ocy-like cells along the ossified tissue (**Fig. 5d**) plus an increase in *Dmp1* in cKO bone-like cells and Ods (**Fig. 5e**). The quantitative data showed near 3-fold increase of the DMP1^+^ Ob-like cells in the cKO crown area (**Figure S9,** P<0.01). Importantly, the immunofluorescent image of Ki67 (**Figure S10a**) and the immunohistochemical staining image of PCNA (**Figure S10b**) showed a great increase in cell proliferation in the cKO bone-like cells (right panels). These changes are statistically significant (p < 0.05; **Figure S10c**). Of note, there were only very minor changes in the molecules described above in the cKO root area (**Figure S11**). Together, these results support the notion that deletion of the *Tgfβr2* in the crown by 3.2* Col1-Cre^ERT2^* greatly downregulated the expression levels of OSX and DSPP (key molecules required for dentinogenesis) and upregulated bone markers such as DMP1 expression, leading to the cell fate switch to the osteogenic lineage and ectopic bone-like tissues.

## Discussion

The close relationship in the development of jawbones and teeth 9-11 and the onset of osteo-dentin (a type of bone-like tissues) in the injured- or the diseased or the gene knockout pulps 1, 3-6, 14 suggest a close linkage between bones and tooth dentin. To precisely demonstrate the cellular lineage linkage between bones and tooth dentin, we investigated whether a switch from dentin-to-bones takes place during postnatal development by specific deletions of the *Tgfβr2* using both early and late Cre-lines combined with cell-lineage tracing approaches. Our key findings in these cKO mice are: **a**. the replacement of odontoblasts by osteoblast/osteocyte-like cells in both crown and root; **b**. a loss of dentin tubules and the switch from dentin matrices to bone matrices; and **c**. the onset of ectopic bone-like tissues in the molar crown chamber. These findings suggested that bones and tooth dentin are possibly derived from overlapping cell lineage at the genetic and molecular levels.

TGF-β signaling pathways are highly conserved in organisms ranging from Nematoda and Arthropoda to Mammalia, and define animal body plans 21, 22 plus a critical role during tooth development 26, 28, 29. These early studies provide a solid foundation for us to test the hypothesis of the genetic and cellular linkage between bones and tooth dentin by targeting this vital signaling pathway combining with cell-lineage tracing approaches. Indeed, both early (targeting the root) and late (targeting the crown) cKO data showed a switch from multiple layers of pre-odontoblasts/odontoblast cells to a single layer of bone-like cells. As a result, the cKO odontoblasts no longer form dentinal tubules and dentin matrix proteins. Instead, these “switched bone cells” form bone-like matrices during root development and the crown response to mechanical loading.

We 30, 37 and others 40, 41 previously reported distinct regulation mechanisms in crown and root development. Now, we showed that removal of *Tgfβr2* by the Gli1^Lin^ in roots or the 3.2 Col1^Lin^ in crown leads to similar defects in both the crown and root, supporting the notion that *Tgfβr2* is one of key master genes controlling entire tooth development. However, the Gli1^Lin^ cKO displayed strong root phenotypes such as an extremely thin root wall with a lack of dentin tubules (**Fig. 1**) whereas the 3.2 Col1^Lin^ cKO exhibited severe defects in the crown, including massive ectopic bone in the crown (**Fig. 4**). We reasoned that the following three factors contribute to these variations: **1**) the Gli1^Lin^ mainly targets the early pulp mesenchymal cell in the root (that defines the cell fate to form dentin tubules during the early developmental period), whereas the 3.2 Col1^Lin^ is predominantly expressed in the crown Ods plus some crown pulp mesenchymal cells, which affects late dentin matrix formation with little impact on dentin tubule formation; **2**) the extremely thin crown wall in the 3.2 Col1^Lin^ cKO crown provides little buffer against the chowing-caused high mechanical loading, which results in dentin fractures; because the cKO pulp mesenchymal cell gains the bone remodeling capability due to the program switch to the osteogenic, massive ectopic ossification takes place (similar to a bone fracture response); and **3**) the root mesenchymal pulp is largely protected by surrounding bones from the impact of the mechanical loading, there is a lack of apparent ectopic ossification.

**Figure 6:**
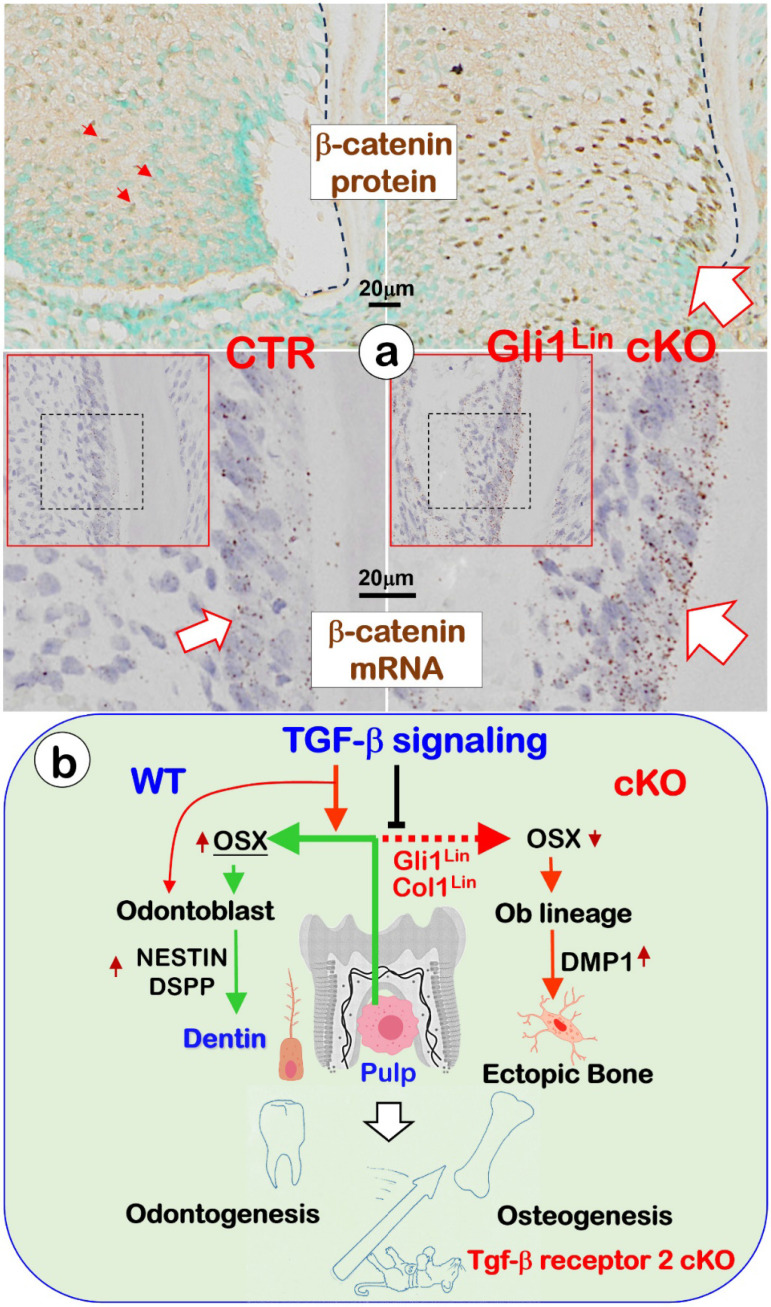
** Changes of β-catenin in the Gli1^Lin^ cKO pulp and the overall working hypothesis. a.** Representative images showed sharp increases of β-catenin in the Gli1^Lin^ cKO pulp/early odontoblasts (Ods) at both protein (right upper panels) and mRNA (right lower panel); and **b**. We propose that TGF-β signaling plays a vital role during odontogenesis by controlling the fate of pulp cells toward the odontogenic pathway, in which Osterix (OSX) positively regulates expressions of NESTIN and DSPP during Od polarization and dentin tubule/matrix formation (see text for details).

Similar to the *Osx* cKO short root phenotype in which there was a great increase in β-catenin expression, the Gli1^Lin^ cKO pulp cells displayed a sharp increase in b-catenin expression at both mRNA and protein levels (**Fig. 6a,** *right panels*). We speculate the increased β-catenin expression (likely due to a sharp reduction of OSX in the Gli1^Lin^ cKO) keeps the pulp cells at an early differentiated status with no further differentiation into odontoblasts 25, 29.

In agreement with the above findings, a separate study reported the ectopic ossification in* Acvr1* cKO molars 1 plus the development of bone-like structure in the *Bmp* receptor 1A cKO crown (data not shown). Furthermore, the onset of ectopic ossification took place in the 3.2 Col1^Lin^ cKO incisor, in which there were high expressions of DMP1, and low levels of DSPP (**Figure S12**). Of note, bone fractures can be self-repaired via their endogenous remodeling mechanisms 1, whereas the fractured teeth caused by traumas cannot repair themselves. These fractured teeth were either surgically extracted or “glued” by using adhesive materials 6 due to a limited remodeling capability. The switch of the odontogenic to the osteogenic program in the cKO molars or incisors raise a possibility to switch a quiescent pulp odontogenic cell pool to osteogenic cells by local deliveries of virus carried *Tgfβr2* knockout constructs in a tooth fracture model. This way, the largely inactive pulp mesenchymal cells will be activated and shifted to bone-like cells after cells taking in the KO construct. As a result, these ectopic bones will likely repair the fractured tooth. If this theoretical idea (that *Tgfβr2* knockout constructs turn pulp mesenchymal cells into bone-like cells) succeeds in the future, it will likely open a new research direction for the treatment of patients with tooth fractures with this approach instead of pulling out fractured teeth in future.

Importantly, we did not observe any sign of a tooth-like phenotype in the *Tgfβr2* cKO jawbone 42 (also see **Fig. 4c**). In other words, removing *Tgfβr2* in bone cells does not switch bones to a tooth-like phenotype, suggesting that the osteogenesis is a default program from which the odontogenic program is derived. We believe that these results provide solid evidence to support the theory of the jawbone being before tooth dentin during evolution 9, 10 at the genetic and molecular levels. Our future goals are to carry on a similar experiment combining more gene knockout models (such as *Bmp* receptor 1a cKO) with the cell lineage tracing approach to support the theory of bones and teeth in the same cell lineage.

In conclusion, our *in vivo* loss of function studies support the following hypothesis (**Fig. 6**): TGF-β signaling plays a vital role during dentinogenesis by controlling the fate of pulp cells toward the odontogenic pathway, in which Osterix (OSX) positively regulates expressions of Nestin and DSPP during Ods polarization and dentinal tubule/matrix formation. Conditionally removing *Tgfβr2* in root pulp cells by the Gli1*-*Cre^ERT2^ line (Gli1^Lin^) or in the crown pulp cells by the 3.2 kb Col1-Cre^ERT2^ line (3.2 Col1^Lin^) leads to a sharp reduction of Nestin and DSPP but a great increase in DMP1. As a result, a switch from odontogenesis to osteogenesis takes place. Current studies also support the theory that both osteogenesis and odontogenesis are likely in the same evolutionary lineage, in which TGF-β signaling controls the fate of odontogenic cells toward the odontogenic via positively controlling OSX, NESTIN and DSPP.

## Supplementary Material

Supplementary figures and table.

## References

[1] Zhang X, Shi C, Zhao H, Zhou Y, Hu Y, Yan G (2019). Distinctive role of ACVR1 in dentin formation: requirement for dentin thickness in molars and prevention of osteodentin formation in incisors of mice. J Mol Histol.

[2] Xie X, Wang J, Wang K, Li C, Zhang S, Jing D (2019). Axin2(+)-Mesenchymal PDL Cells, Instead of K14(+) Epithelial Cells, Play a Key Role in Rapid Cementum Growth. J Dent Res.

[3] Takuma S, Yanagisawa T, Lin WL (1977). Ultrastructural and microanalytical aspects of developing osteodentin in rat incisors. Calcif Tissue Res.

[4] Holmstedt JO, McClugage SG Jr, Clark JS, Guevara MJ (1977). Osteodentin formation in continuously erupting teeth of guinea pigs. J Dent Res.

[5] Karim AC, Eddy EL (1984). A light and electron microscopic study of osteodentin formation in the rat incisor after adriamycin administration. Am J Anat.

[6] Karim AC, Pylypas SP (1986). Osteodentin formation in rat incisor as visualized by radioautography after 3H-proline administration. Anat Rec.

[7] Sansom IJ, Smith MP, Armstrong HA, Smith MM (1992). Presence of the earliest vertebrate hard tissue in conodonts. Science.

[8] Donoghue PC, Forey PL, Aldridge RJ (2000). Conodont affinity and chordate phylogeny. Biol Rev Camb Philos Soc.

[9] Murdock DJ, Dong XP, Repetski JE, Marone F, Stampanoni M, Donoghue PC (2013). The origin of conodonts and of vertebrate mineralized skeletons. Nature.

[10] Janvier P (2013). Palaeontology: Inside-out turned upside-down. Nature.

[11] Donoghue PC, Rucklin M (2016). The ins and outs of the evolutionary origin of teeth. Evol Dev.

[12] Ruch JV (1995). Tooth crown morphogenesis and cytodifferentiations: candid questions and critical comments. Connect Tissue Res.

[13] Larmas M (2008). Pre-odontoblasts, odontoblasts, or "odontocytes". J Dent Res.

[14] Shi P, Xie X, Xu C, Wu Y, Wang J (2023). Activation of Wnt signaling in Axin2(+) cells leads to osteodentin formation and cementum overgrowth. Oral Dis.

[15] Huang XF, Chai Y (2012). Molecular regulatory mechanism of tooth root development. Int J Oral Sci.

[16] Li J, Parada C, Chai Y (2017). Cellular and molecular mechanisms of tooth root development. Development.

[17] Zeichner-David M, Oishi K, Su Z, Zakartchenko V, Chen LS, Arzate H (2003). Role of Hertwig's epithelial root sheath cells in tooth root development. Dev Dyn.

[18] Butler WT, Ritchie H (1995). The nature and functional significance of dentin extracellular matrix proteins. Int J Dev Biol.

[19] Kawashima N, Okiji T (2016). Odontoblasts: Specialized hard-tissue-forming cells in the dentin-pulp complex. Congenit Anom (Kyoto).

[20] Wang J, Feng JQ (2017). Signaling Pathways Critical for Tooth Root Formation. J Dent Res.

[21] Patterson GI, Padgett RW (2000). TGF beta-related pathways. Roles in Caenorhabditis elegans development. Trends Genet.

[22] Zinski J, Tajer B, Mullins MC (2018). TGF-βeta Family Signaling in Early Vertebrate Development. Cold Spring Harb Perspect Biol.

[23] Toyono T, Nakashima M, Kuhara S, Akamine A (1997). Temporal changes in expression of transforming growth factor-beta superfamily members and their receptors during bovine preodontoblast differentiation *in vitro*. Arch Oral Biol.

[24] Zhao H, Oka K, Bringas P, Kaartinen V, Chai Y (2008). TGF-βeta type I receptor Alk5 regulates tooth initiation and mandible patterning in a type II receptor-independent manner. Dev Biol.

[25] Zhang R, Lin J, Liu Y, Yang S, He Q, Zhu L (2021). Transforming Growth Factor-beta Signaling Regulates Tooth Root Dentinogenesis by Cooperation With Wnt Signaling. Front Cell Dev Biol.

[26] Oka S, Oka K, Xu X, Sasaki T, Bringas P Jr, Chai Y (2007). Cell autonomous requirement for TGF-βeta signaling during odontoblast differentiation and dentin matrix formation. Mech Dev.

[27] Kikuchi H, Amano H, Yamada S (2001). Putative role of basement membrane for dentinogenesis in the mesenchyme of murine dental papillae *in vitro*. Cell Tissue Res.

[28] Ko SO, Chung IH, Xu X, Oka S, Zhao H, Cho ES (2007). Smad4 is required to regulate the fate of cranial neural crest cells. Dev Biol.

[29] Li J, Huang X, Xu X, Mayo J, Bringas P Jr, Jiang R (2011). SMAD4-mediated WNT signaling controls the fate of cranial neural crest cells during tooth morphogenesis. Development.

[30] Wang J, Massoudi D, Ren Y, Muir AM, Harris SE, Greenspan DS (2017). BMP1 and TLL1 Are Required for Maintaining Periodontal Homeostasis. J Dent Res.

[31] Wang J, Jiang Y, Xie X, Zhang S, Xu C, Zhou Y (2022). The identification of critical time windows of postnatal root elongation in response to Wnt/beta-catenin signaling. Oral Dis.

[32] Feng JQ, Ward LM, Liu S, Lu Y, Xie Y, Yuan B (2006). Loss of DMP1 causes rickets and osteomalacia and identifies a role for osteocytes in mineral metabolism. Nat Genet.

[33] Wang F, Flanagan J, Su N, Wang LC, Bui S, Nielson A (2012). RNAscope: a novel *in situ* RNA analysis platform for formalin-fixed, paraffin-embedded tissues. J Mol Diagn.

[34] Jing J, Hinton RJ, Mishina Y, Liu Y, Zhou X, Feng JQ (2014). Critical role of Bmpr1a in mandibular condyle growth. Connect Tissue Res.

[35] Zhao H, Feng J, Seidel K, Shi S, Klein O, Sharpe P (2014). Secretion of shh by a neurovascular bundle niche supports mesenchymal stem cell homeostasis in the adult mouse incisor. Cell Stem Cell.

[36] Feng J, Jing J, Li J, Zhao H, Punj V, Zhang T (2017). BMP signaling orchestrates a transcriptional network to control the fate of mesenchymal stem cells in mice. Development.

[37] Zhang H, Jiang Y, Qin C, Liu Y, Ho SP, Feng JQ (2015). Essential role of osterix for tooth root but not crown dentin formation. J Bone Miner Res.

[38] Sreenath T, Thyagarajan T, Hall B, Longenecker G, D'Souza R, Hong S (2003). Dentin sialophosphoprotein knockout mouse teeth display widened predentin zone and develop defective dentin mineralization similar to human dentinogenesis imperfecta type III. J Biol Chem.

[39] Maes C, Kobayashi T, Selig MK, Torrekens S, Roth SI, Mackem S (2010). Osteoblast precursors, but not mature osteoblasts, move into developing and fractured bones along with invading blood vessels. Dev Cell.

[40] Kim TH, Bae CH, Lee JY, Lee JC, Ko SO, Chai Y (2015). Temporo-spatial requirement of Smad4 in dentin formation. Biochem Biophys Res Commun.

[41] Ahn YH, Kim TH, Choi H, Bae CH, Yang YM, Baek JA (2015). Disruption of Tgfβr2 in odontoblasts leads to aberrant pulp calcification. J Dent Res.

[42] Xu C, Xie X, Zhao H, Wu Y, Wang J, Feng JQ (2021). TGF-Beta Receptor II Is Critical for Osteogenic Progenitor Cell Proliferation and Differentiation During Postnatal Alveolar Bone Formation. Front Physiol.

