# Supplementary material for: Bone or Tooth dentin: The TGF-β signaling is the key
Source: Int J Biol Sci. 2024 Jun 24;20(9):3557–69. doi: 10.7150/ijbs.97206 (PMC11234226; doi:10.7150/ijbs.97206)
Supplement: Supplementary file 1 — Supplementary figures and table. [file ijbsv20p3557s1.pdf]

Table S1. RNAscope scoring categories

Staining score	Microscope Objective Scoring (Dots number, 40X)
0	0-1 dot/10 cells
1	1-3 dots/cell
2	4-10 dots/cell with very few dot clusters
3	>10 dots/cell with less than 10% positive cells having dot clusters
4	>10 dots/cell with more than 10% but less than 30 % positive cells having dot clusters
5	>10 dots/cell with more than 30% but less than 50% positive cells having dot clusters
6	>10 dots/cell with more than 50% but less than 70% positive cells having dot clusters
7	>10 dots/cell with more than 70% but less than 90% positive cells having dot clusters
8	>10 dots/cell with more than 90% positive cells having dot clusters

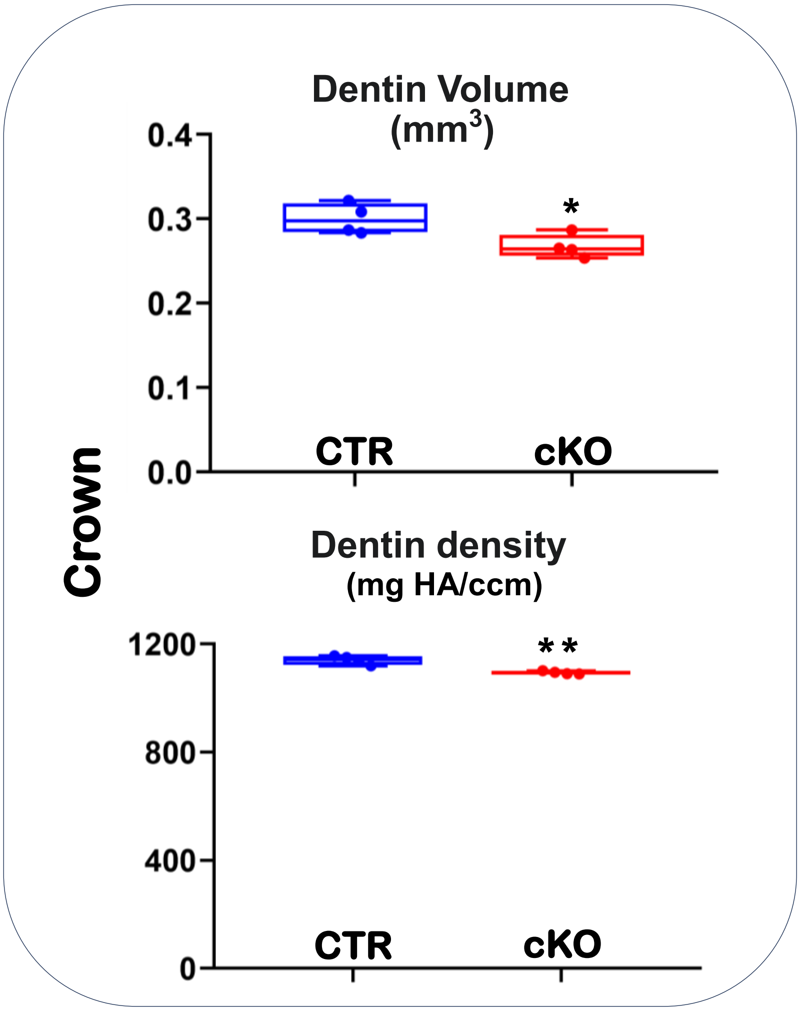

Figure S1. Removal of *Tgfβr2* caused minor changes in the Gli1^{Lin} cKO crown dentin volume and dentin density. Micro-CT analyses displayed a moderate reduction in the crown dentin volume by ~10% (top panel; n =4; *P<0.05), and a minor decrease in the crown dentin density by ~5% (n =4; *P< 0.05; **P< 0.01).

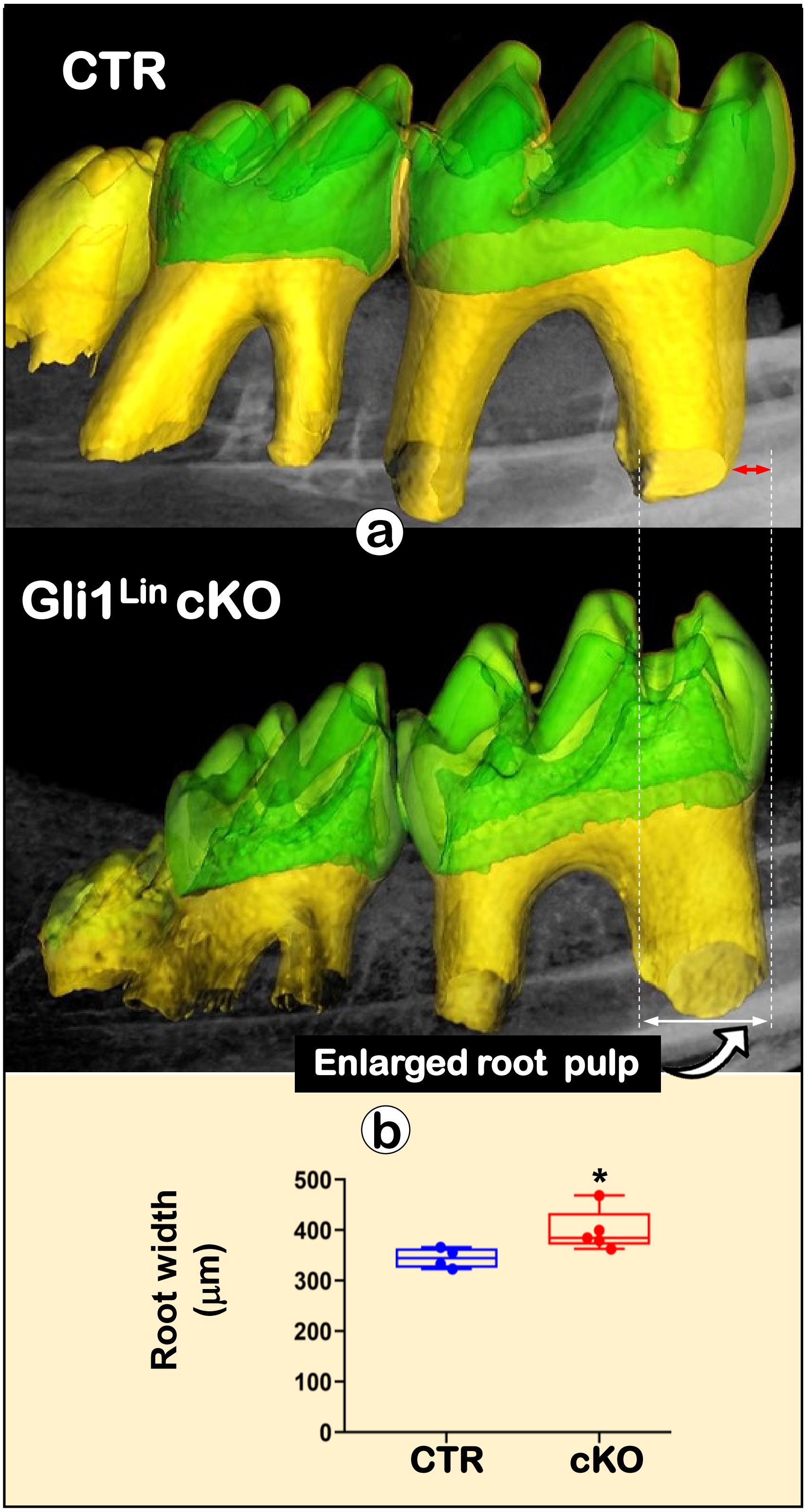

Figure S2. Removal of *Tgfβr2* led to enlarged root canal in the Gli1^{Lin} cKO. a. Three-dimensional reconstruction of μCT images from CTR and Gli1^{Lin} cKO mandibles were performed with Imaris 9.0 (Bitplane); and **b**. The quantitation of distal root width of the Gli1^{Lin} cKO vs CTR was analyzed using ImageJ (n = 4~5; *P< 0.05)

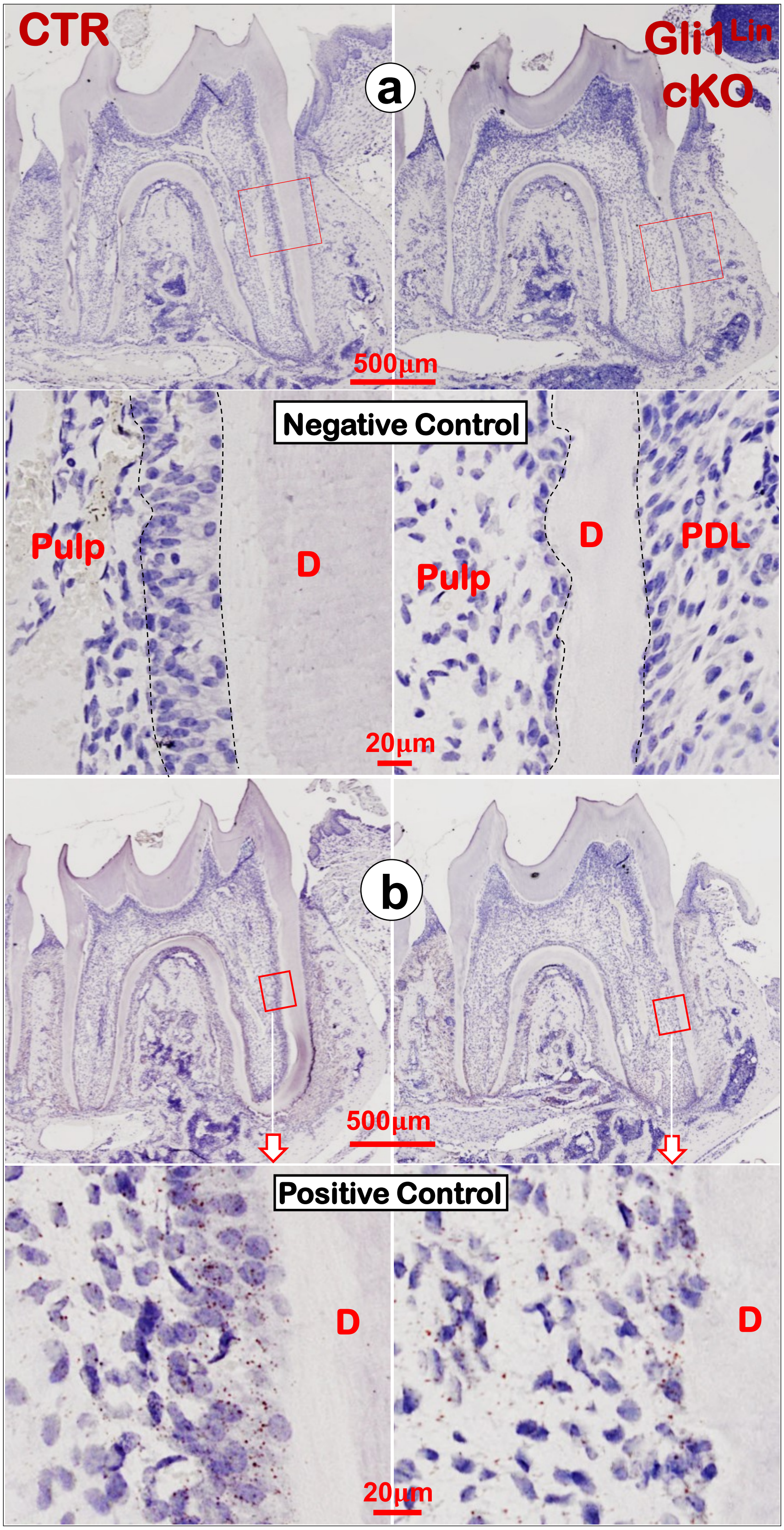

Figure S3. Tests of negative and positive controls using the RNAscope technique. a. Five-µm-thick sections of decalcified paraffin-embedded molar samples from the age-matched control and Gli1^{Lin} cKO were used for RNAscope assay, in which a negative control probe was used and displayed no signal in both the CTR and Gli1^{Lin} cKO samples. **b**. The test of the positive control probe displayed a similar signal pattern in the Ods (*left panel*) and in the Ob-like cells (*right panel*). PDL, periodontal ligament; D, dentin.

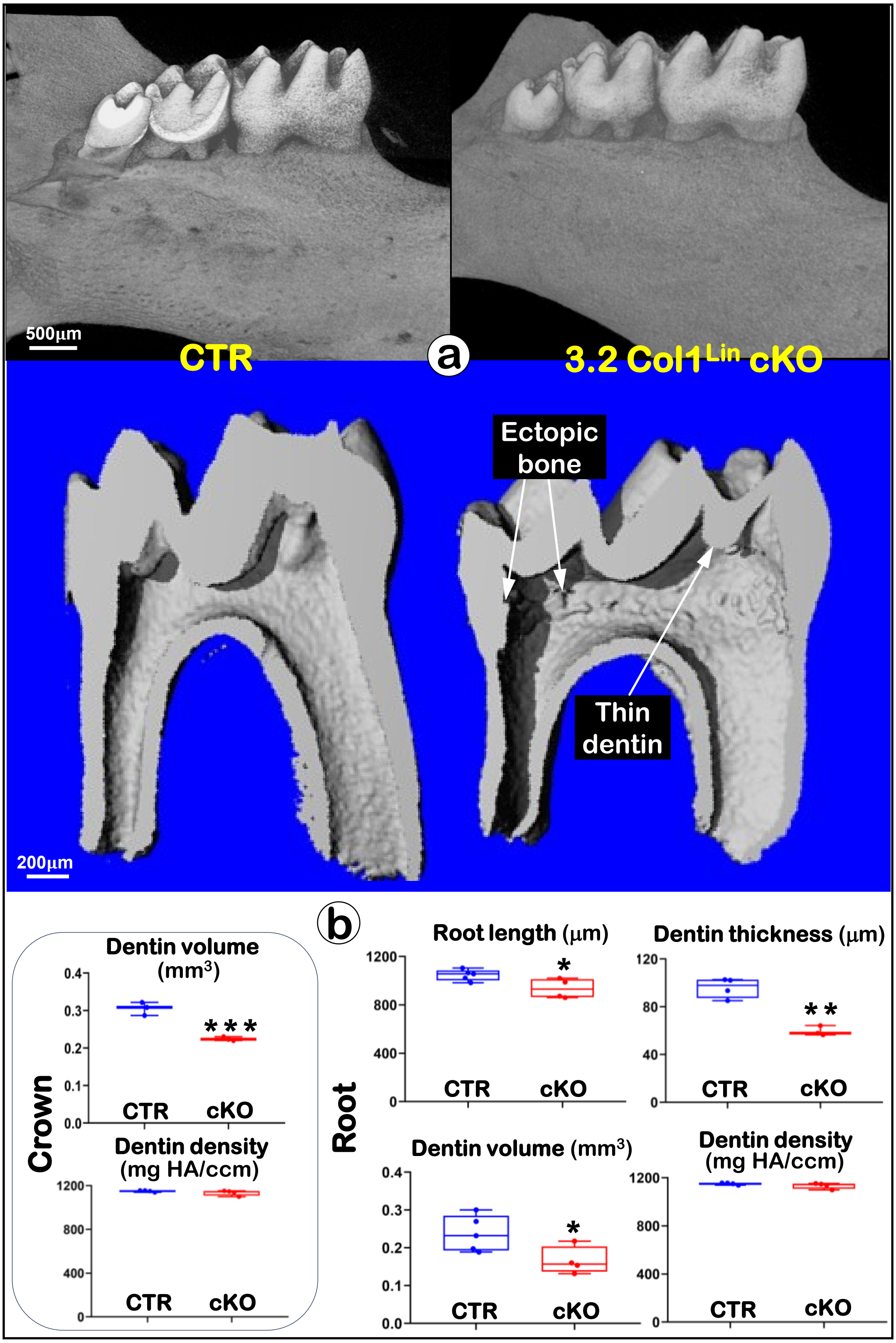

Figure S4. Quantitation of 3.2 Col1^{Lin} cKO molars. a. Representative µCT 3D images of mandibles and first molars, in which the 3.2 Col1^{Lin} cKO crown was thin. The pulp space was enlarged and roots was slightly thin and short (*right lower panel*); and **b.** Quantitative analyses of µCT changes of molar crown and root including dentin volume, dentin density, root length and dentin thickness (n=3~5; *P< 0.05; **P< 0.01 ; ***P< 0.001).

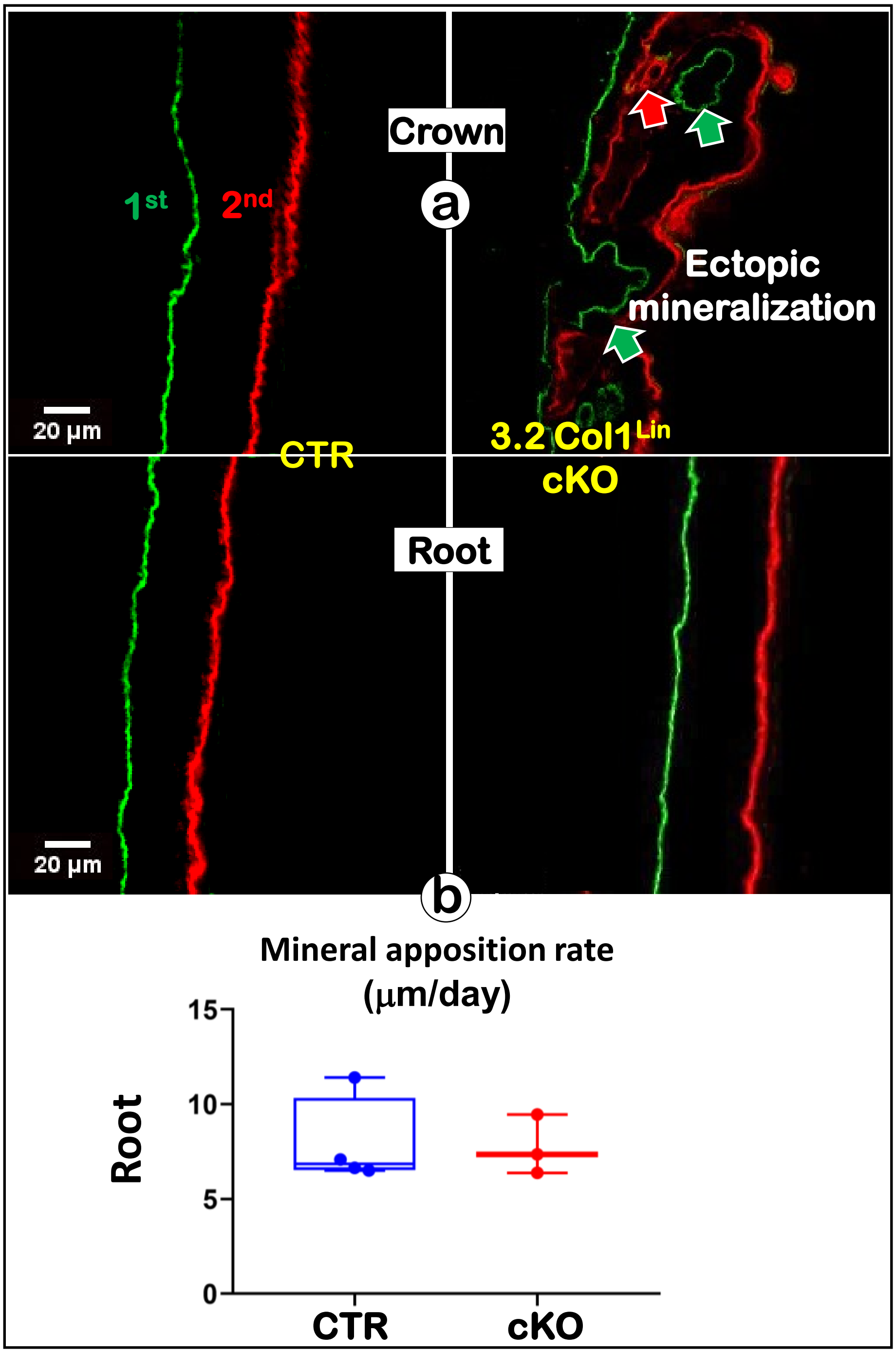

Figure S5. Conditional knockout of *Tgfβr2* in the 3.2 Col1^{Lin} led to ectopic ossification in the 3.2 Col1^{Lin} cKO crown. a. The representative double labeling data showed massive ectopic ossification in the cKO crown (*right panel; green and red arrows*); and **b**. The double-labeling data of the roots displayed no obvious difference between the control (*top left panel)* and the cKO root (*top right panel).* There was no statistical significance between the control and cKO in the mineral apposition rate of root dentin (*lower panel).* (n =3~4; $p > 0.05$)

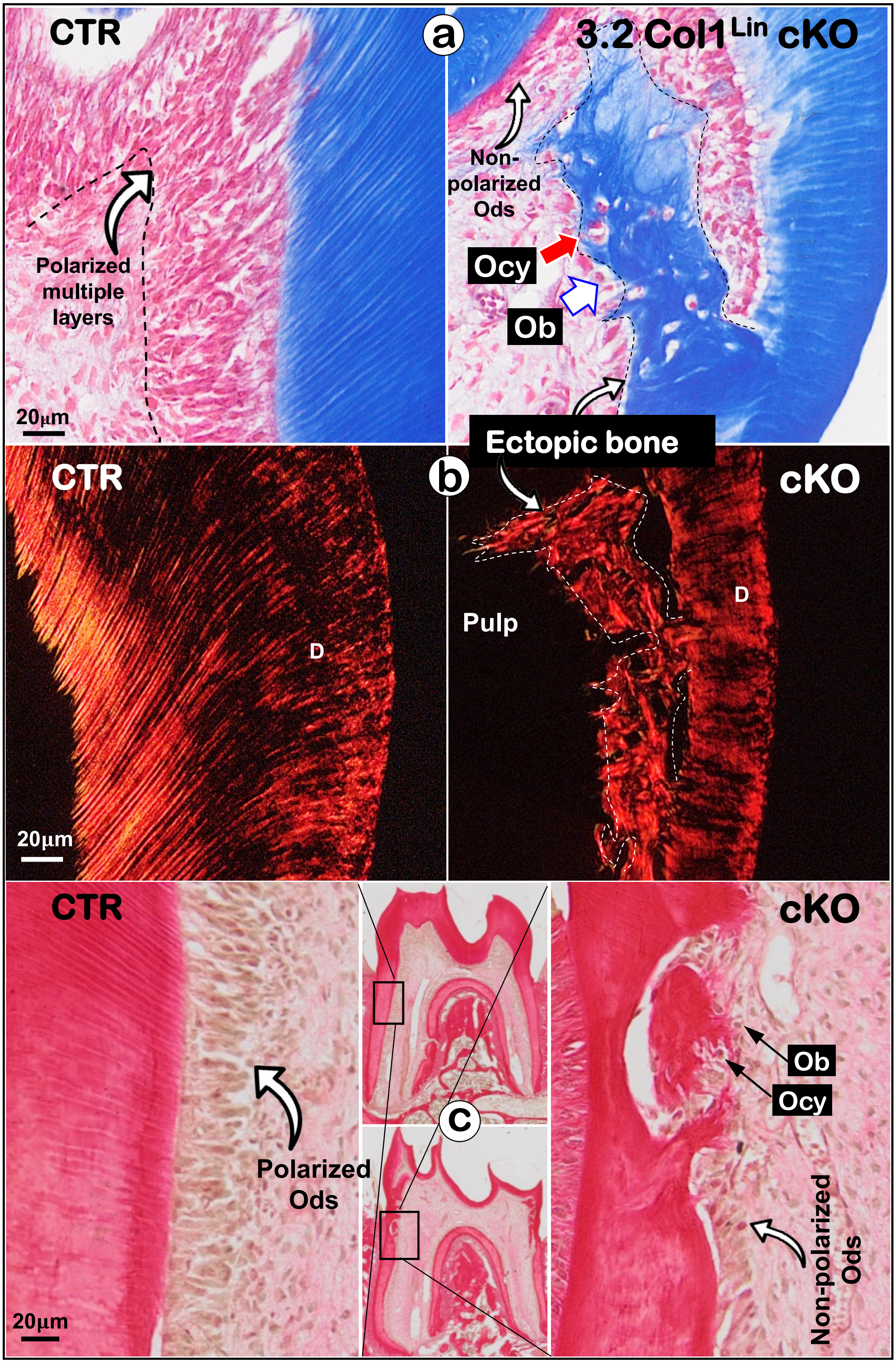

Figure S6. Ectopic ossification formed in the 3.2 Col1^{Lin} cKO crown with osteocyte-like cells embedded inside. **a**. Masson's Trichrome staining images showed a thin crown and non-polarized osteoblast (Ob) -like cells plus ectopic ossification with one layer of Obs along its surface and osteocyte (Ocy) -like cells buried in its matrix; **b**. Polarized light microscope images revealed ossification and bone-like matrix in the 3.2 Col1^{Lin} cKO crown; and **c.** Sirius Red staining showed both Ob and Ocy like cells surrounding the cKO-caused ectopic bone (right panel). Ocy, osteocyte; Ob, osteoblast; D, dentin.

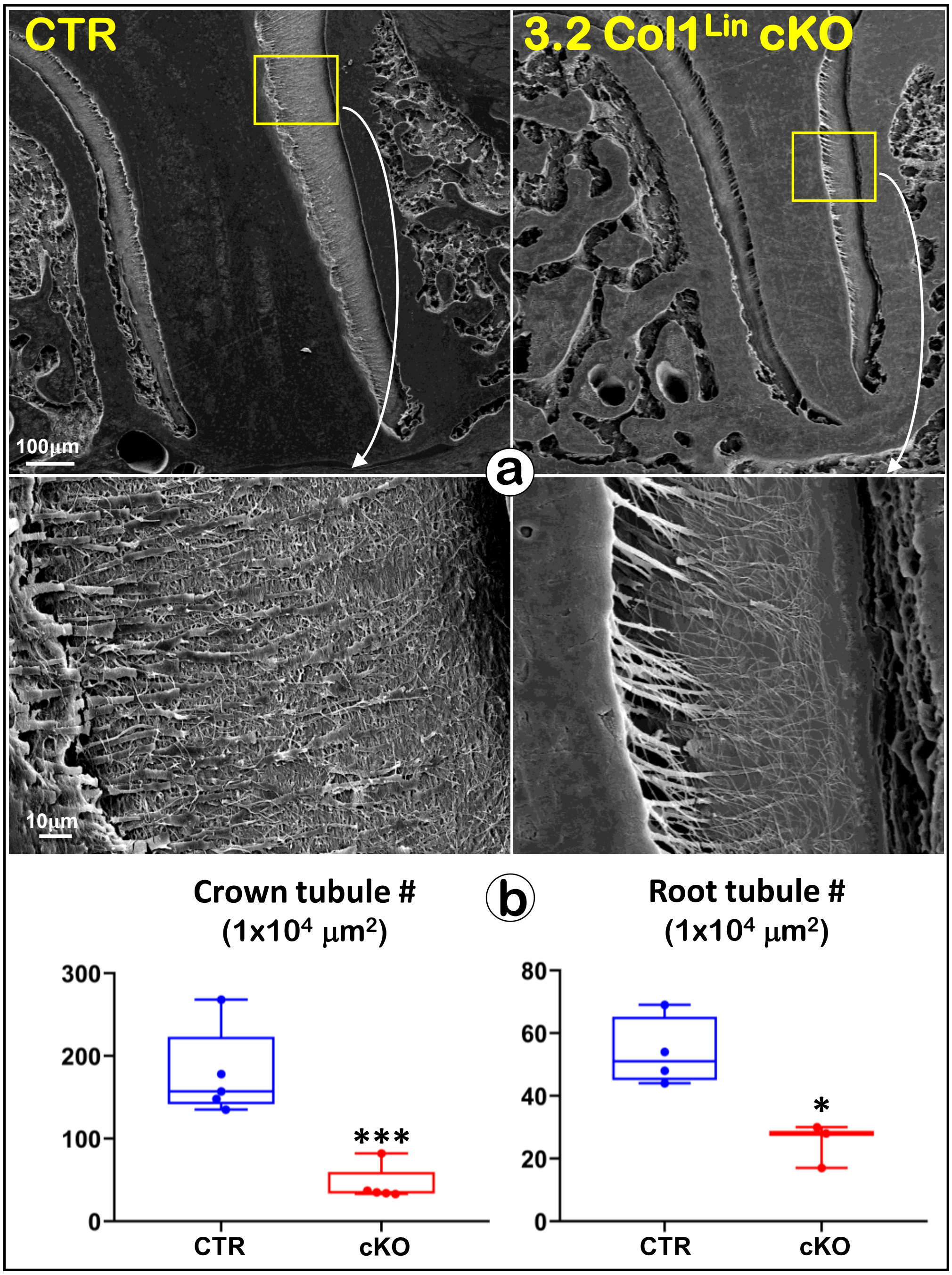

Figure S7. Conditional knockout of *Tgfβr2* caused moderate changes in both crown and root dentinal tubule numbers in the 3.2 Col1^{Lin} cKO. a. Representative acid-etched SEM images displayed a moderate reduction of crown and root dentinal tubules in the 3.2 Col1^{Lin} cKO (*right panels*); and **b**. Statistic analyses showed that differences between groups are statistically significant (n=4~5; *P < 0.05; ***P < 0.001).

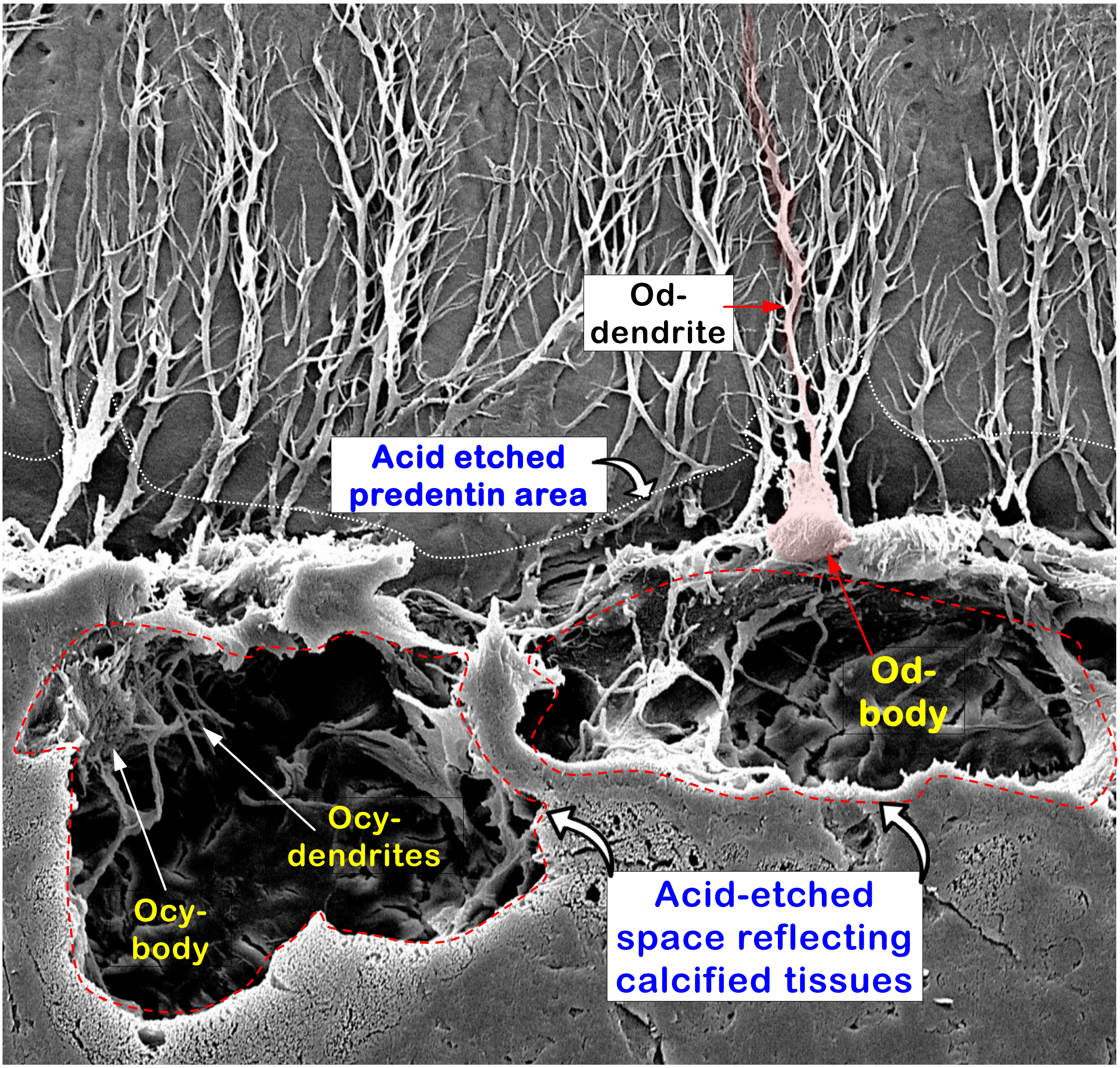

Figure S8. Acid-etched SEM images of the ectopic bone in the 3.2 Col1^{Lin} cKO crown. Methylmethacrylate (MMA) embedded molar blocks were used for SEM analyses as previously described. The surfaces of MMA-embedded crowns were re-polished and acid-etched with 37% phosphoric acid for 2-10 seconds, followed by two 20-minute washes with 5% sodium hypochlorite. Subsequently, the blocks were washed, dried, and coated with gold/palladium prior to SEM imaging. The SEM image displayed an osteocyte (Ocy) and its dendrites surrounded by an empty space etched by acid removal of mineral and matrix tissue, whereas an Od cell and its single dendrite were artificially colored in yellow. Of note, this Od was buried in the predentin area, which was removed by acid treatment. The above data suggested that an Ocy-like cell is buried in the ectopic bone. Od, odontoblast.

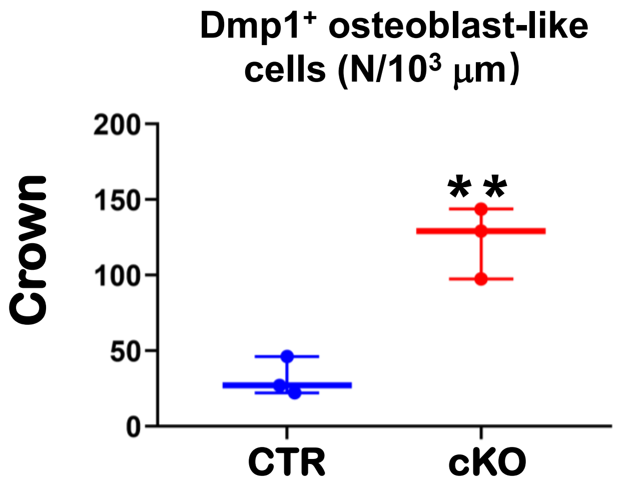

Figure S9. More than 3-fold increases in the *Dmp1*$^{+}$ osteoblast-like cell number in 3.2 Col1^{Lin} cKO crown based on the RNAscope assay. (n =3; **P< 0.01).

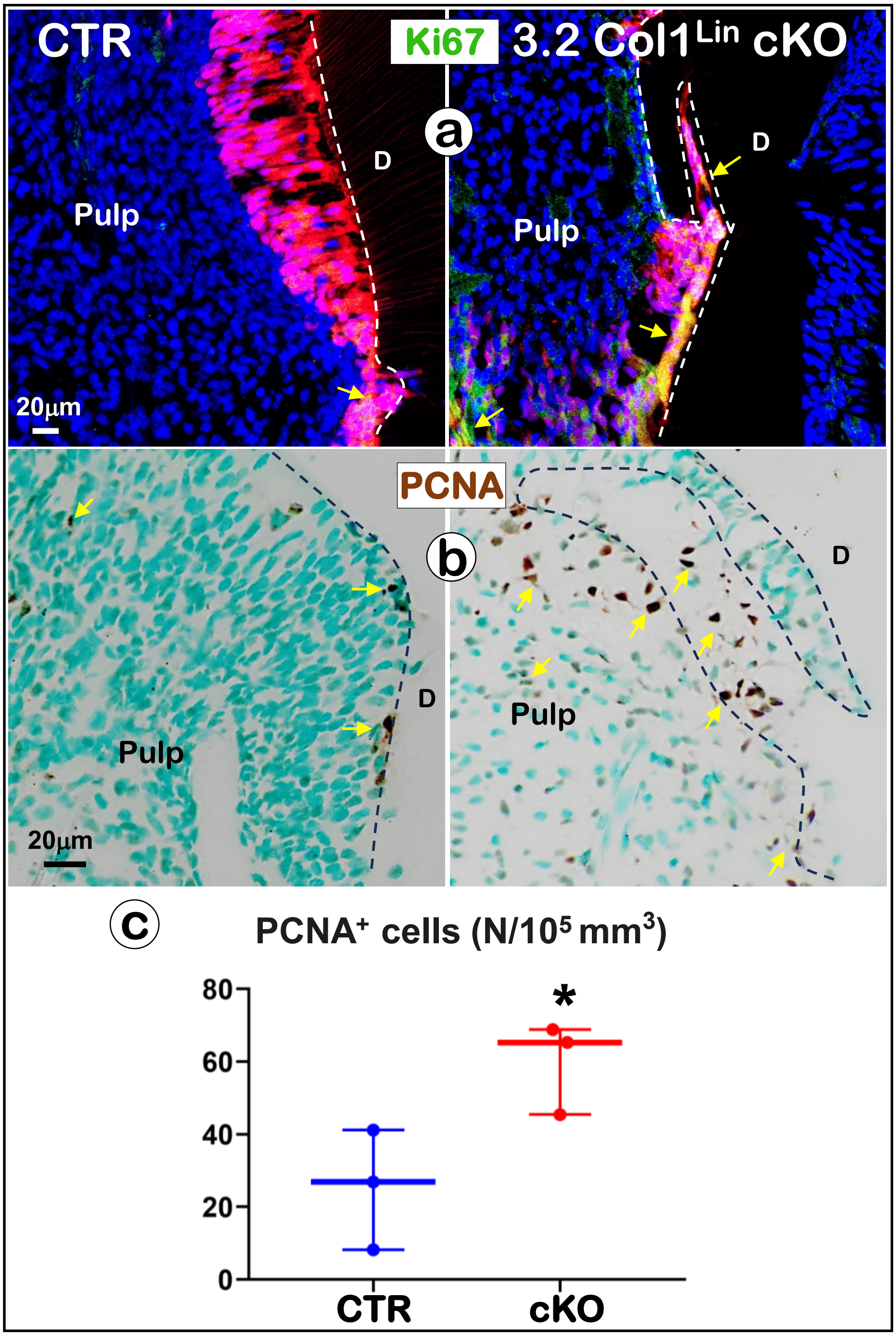

Figure S10. Removal of *Tgfβr2* caused significant increases in cell proliferation of bone-like cells in the 3.2 Col1^{Lin}-cKO crown. a. Representative immunofluorescent images of Ki67 (green color) in the background of 3.2 Col1^{Lin}-tdTomato (red) displayed an increase in flat bone-like cells that are Ki67^{+} (yellow color due to the overlap of green and red signals); **b**. Representative immunostaining images of PCNA (brown color) showed a significant increase in 3.2 Col1^{Lin} cKO bone-like cells (*right panel*); and **c**. The quantitative data displayed more than 2-fold increase in the 3.2 Col1^{Lin} cKO (n=3; *$p < 0.05$). D, dentin.

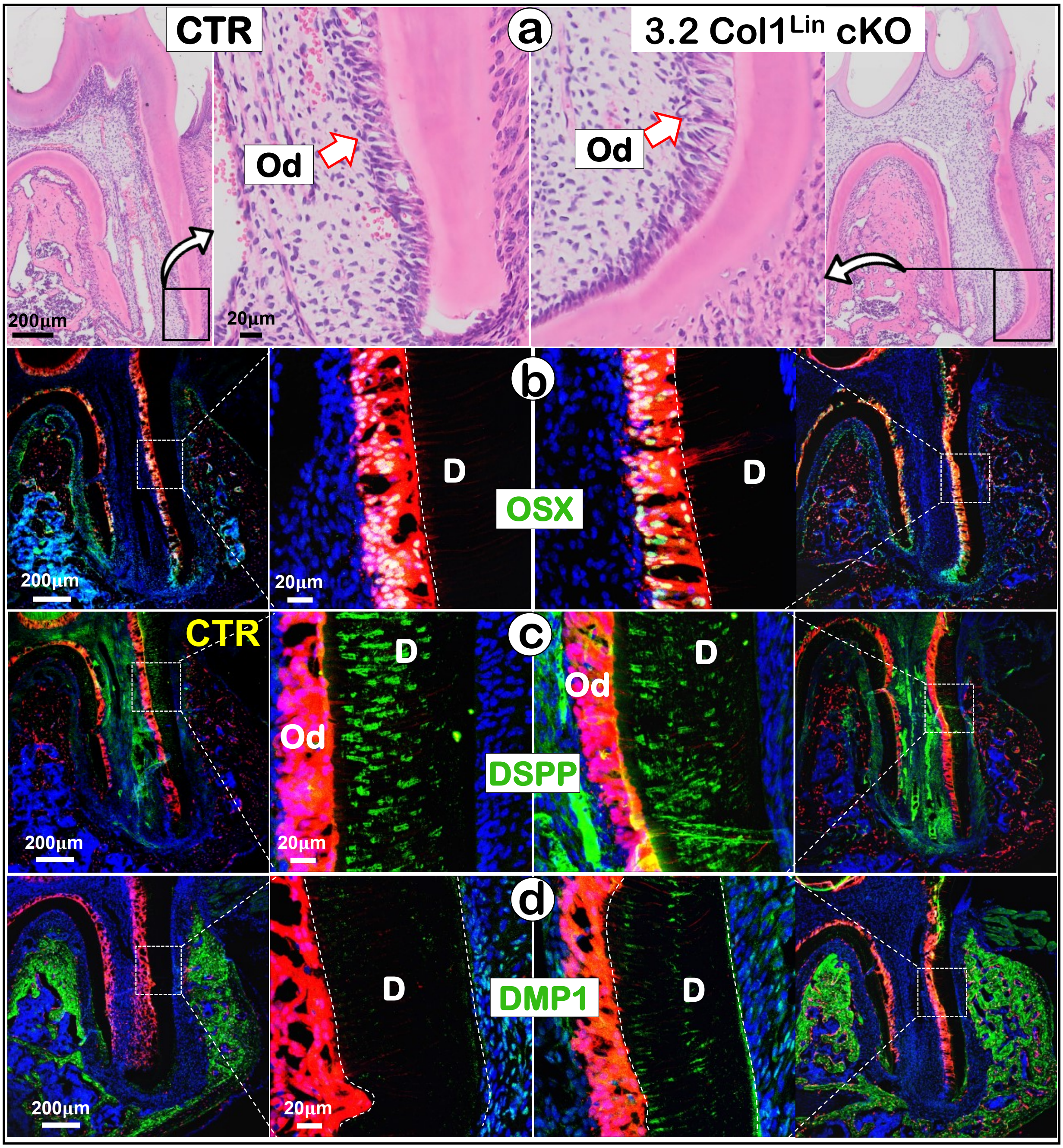

Figure S11. There are only moderate changes in cellular structures and molecular expression profiles in the 3.2 $Col1^{Lin}$ cKO roots. a. Representative H&E images displayed multi-layer polarized odontoblasts (Ods) in both the CTR (*left panel*) and the 3.2 $Col1^{Lin}$ cKO root (*right panel*); and **b-e.** Immunofluorescence staining images showed no apparent changes of the following markers in the 3.2 $Col1^{Lin}$ cKO root: OSX (**b**), DSPP (**c**), and DMP1 (**d**) in the 3.2 $Col1^{Lin}$ cKO root dentin. Od, odontoblasts; D, dentin.

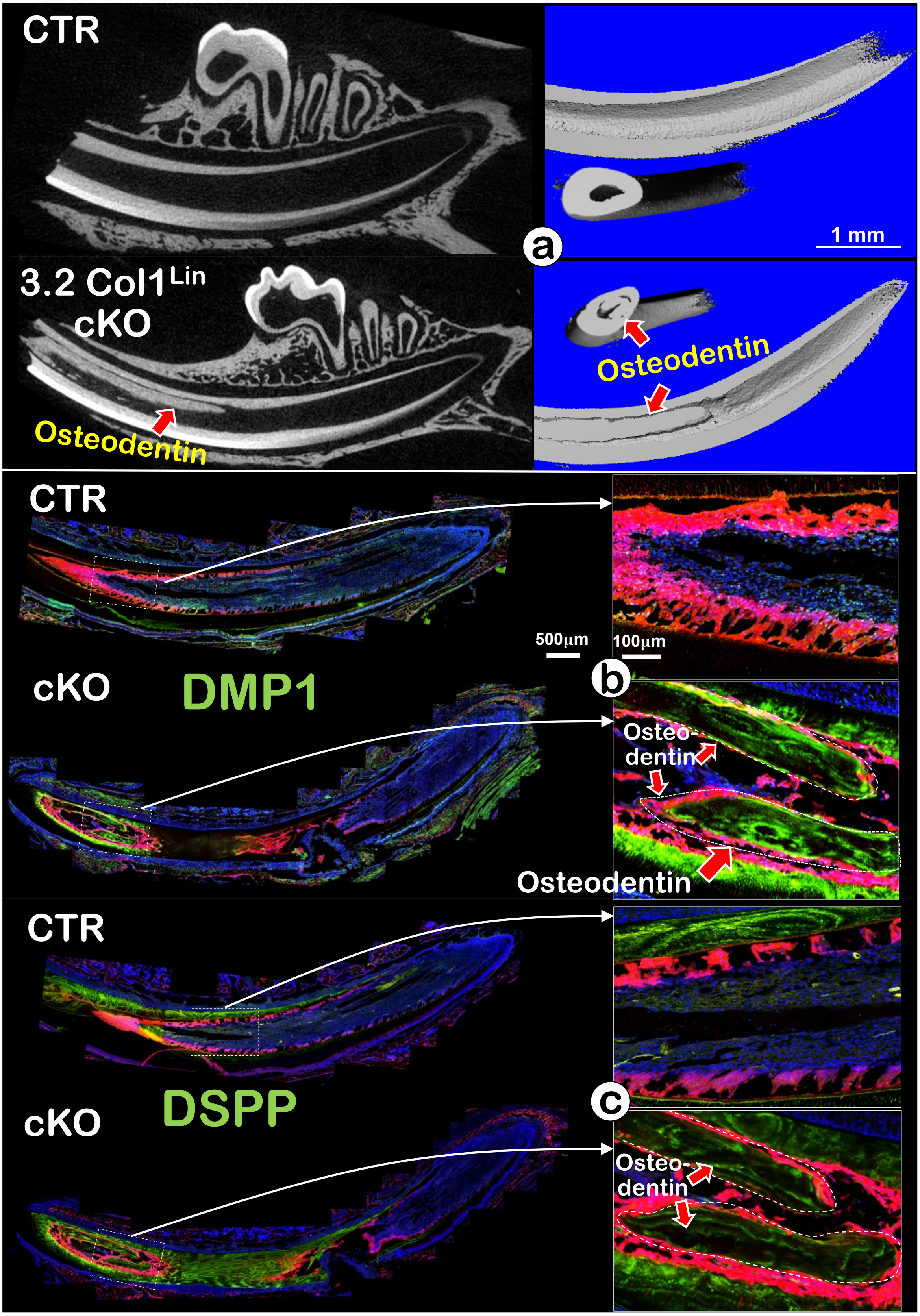

Figure S12. The formation of ectopic ossification in the 3.2 Col1^{Lin} cKO incisor. **a**. The µCT images of 2D (left panels) and 3D (right panels) showed the formation of ectopic ossification structure (osteo-dentin; lower right panel); **b**. Confocal images of immunofluorescent staining revealed high levels of DMP1 in the ossification area of the 3.2 Col1^{Lin} cKO incisor (lower right panel); and **c**. Confocal images of immunofluorescent staining revealed a low level of DSPP in the ossification site of the 3.2 Col1^{Lin} cKO incisor (lower right panel).
